# Graphene Oxide (GO) for the Treatment of Bone Cancer: A Systematic Review and Bibliometric Analysis

**DOI:** 10.3390/nano14020186

**Published:** 2024-01-13

**Authors:** Lemy Vanessa Barba-Rosado, Domingo César Carrascal-Hernández, Daniel Insuasty, Carlos David Grande-Tovar

**Affiliations:** 1 Grupo de Investigación en Fotoquímica y Fotobiología, Programa de Química, Facultad de Ciencias Básicas, Universidad del Atlántico, Puerto Colombia 081008, Colombia; lbarba@mail.uniatlantico.edu.co (L.V.B.-R.); dcarrascal@est.uniatlantico.edu.co (D.C.C.-H.); 2 Departamento de Química y Biología, División de Ciencias Básicas, Universidad del Norte, Km 5 Vía Puerto Colombia, Barranquilla 081007, Colombia; insuastyd@uninorte.edu.co

**Keywords:** bone cancer, bibliometric analysis, graphene oxide, reduced graphene oxide, osteosarcoma

## Abstract

Cancer is a severe disease that, in 2022, caused more than 9.89 million deaths worldwide. One worrisome type of cancer is bone cancer, such as osteosarcoma and Ewing tumors, which occur more frequently in infants. This study shows an active interest in the use of graphene oxide and its derivatives in therapy against bone cancer. We present a systematic review analyzing the current state of the art related to the use of GO in treating osteosarcoma, through evaluating the existing literature. In this sense, studies focused on GO-based nanomaterials for potential applications against osteosarcoma were reviewed, which has revealed that there is an excellent trend toward the use of GO-based nanomaterials, based on their thermal and anti-cancer activities, for the treatment of osteosarcoma through various therapeutic approaches. However, more research is needed to develop highly efficient localized therapies. It is suggested, therefore, that photodynamic therapy, photothermal therapy, and the use of nanocarriers should be considered as non-invasive, more specific, and efficient alternatives in the treatment of osteosarcoma. These options present promising approaches to enhance the effectiveness of therapy while also seeking to reduce side effects and minimize the damage to surrounding healthy tissues. The bibliometric analysis of photothermal and photochemical treatments of graphene oxide and reduced graphene oxide from January 2004 to December 2022 extracted 948 documents with its search strategy, mainly related to research papers, review papers, and conference papers, demonstrating a high-impact field supported by the need for more selective and efficient bone cancer therapies. The central countries leading the research are the United States, Iran, Italy, Germany, China, South Korea, and Australia, with strong collaborations worldwide. At the same time, the most-cited papers were published in journals with impact factors of more than 6.0 (2021), with more than 290 citations. Additionally, the journals that published the most on the topic are high impact factor journals, according to the analysis performed, demonstrating the high impact of the research field.

## 1. Introduction

Cancer is a non-communicable disease that consists of the uncontrolled growth of cells with genetic defects or alterations [1,2]. These alterations can be generated by various causes (prolonged exposure to radiation, chemical substances, and congenital disorders caused by consuming carcinogens, among others). They can develop in any body part, seriously affecting organs and tissues through metastatic spread [3]. Cancer is a global public health problem and the second leading cause of premature death in many countries [4,5]. The American Cancer Society (SAC) estimated that, for 2023, about 1,958,310 new cancer cases in the United States (with about 3970 new cases of bone and joint cancer) and about 609,820 cases of death (around 2140 cases of death associated with bone cancer) would occur [6].

There are several types of bone cancer. Among them, osteosarcoma is a type of bone cancer mainly affecting children, adolescents, and young adults, seriously compromising long bones such as the limbs and pelvis [7]. In addition, there is a registry of Ewing’s sarcoma and chondrosarcoma, which are types of cancer associated with significant morbidity and mortality [8]. However, various approaches have been reported for the treatment of this disease. Some employ functional materials commonly used in bone tissue engineering [9], such as Paclitaxel combined with platinum (known as gemcitabine) for the treatment of malignant neoplasms in bone tissues [10]. However, many conventional drugs have been shown to cause conditions such as hypothyroidism [11].

Similarly, the frequent use of taxanes for synthesizing and designing anti-cancer drugs presents low selectivity and physiological solubility, generating renal and hepatic deficiencies [12]. On the other hand, although recent research has shown that using graphene oxide (GO) and reduced graphene oxide (rGO) is promising for the treatment of bone cancer, more research is still required to increase its cytotoxicity against osteosarcoma and other types of cancer, maintaining its biocompatibility in non-target cells [13]. GO and rGO are versatile nanomaterials used for gene delivery and drugs, and are often used in tissue engineering [14]. These structures have a large surface area that facilitates their chemical functionalization. In addition, they present good thermal conduction that allows for localized photothermal therapy. These properties make it attractive for the development of cancer therapies [15]. Recently, photodynamic therapy, photothermal therapy, and nanocarriers have been techniques that have garnered interest in treating osteosarcoma [16]. Photodynamic therapy consists of photosensitizers that, when irradiated with light, usually near-infrared NIR light, produce reactive oxygen species and singlet oxygen that affect tumor cells, generating apoptosis or cell necrosis [17].

Photothermal therapy is based on optical light absorption by photothermal agents such as silver (Ag), gold (Au), and carbon-based nanoparticles. The absorbed light is converted into heat, increasing the cancer cells’ temperature and generating cell death by necrosis and the degradation of enzymes [18]. 

Nanocarriers, on the other hand, are drug-loading systems that are incorporated into organic or inorganic structures, modifying their characteristics such as composition, shape, and surface to improve the therapeutic properties of drugs, their efficiency, solubility, controlled drug release at specific target sites, minimizing side effects [19]. When these therapies are functionalized, they potentiate the therapeutic effects, increasing solubility and efficacy [20,21].

In this sense, the functionalization of these nanomaterials can significantly increase their selectivity and cytotoxicity in cancer cells. For example, a novel photothermal functional GO-based drug delivery system applying near-infrared (NIR-IR) was able to target the mitochondria of cancer cells, demonstrating synergistic and targeted phototherapy. In this study, (4-carboxybutyl)triphenylphosphonium bromide (TPP) was conjugated with PEGylated graphene oxide nanosheets modified with polyethyleneimine (PEI) and loaded with indocyanine green (TPP-PPG@ICG) which allowed the therapy’s synergistic photodynamic and photothermal treatment to overcome osteosarcoma resistance to conventional drugs [22]. In addition, the synthesis of a complex functionalized through the photosensitive agents 2-(1-hexyloxyethyl)-2-divinyl pyropheophorbide-alpha (HPPH), cell-penetrating peptides (CPP), epirubicin (EPI), and pegylated GO (HPPH) has been reported (EPI-CPP-pGO), which proved to be an excellent inhibitor of osteosarcoma growth in vitro [23]. Likewise, the non-covalent association of a maleimide-dopamine adduct (dopa-MAL) with rGO and subsequent click chemistry reaction via the thiol-ene Michael of a synthetic cyclic peptide (RGD), for the recognition of cancer cell integrins and its load with a drug used in cancer chemotherapy, doxorubicin DOX, was reported [24].

This systematic review highlights GO and rGO’s attributes in designing therapies against bone cancer. The various alternatives that will allow for the design of efficient treatments through the chemical functionalization of GO are also presented, which will provide essential elements to reduce the numbers of deaths and diseases derived from osteosarcoma and its traditional non-selective treatments, aligned with the objective of sustainable development goal 3 (SDG 3): “health and well-being for all”, and goal 3b “Support research and development of vaccines and medicines for communicable and non-communicable diseases that primarily affect developing countries…”. On the other hand, the bibliometric analysis of the 948 documents extracted (see the supporting information) allowed information to be obtained on the trends in publications, authors, and affiliations that are contributing the most to and updating the field of GO and rGO’s anti-cancer applications against bone cancer. This approach is essential for the biomedical, clinical, and scientific fields, providing information that allows for mitigating the side effects of conventional treatments and increasing the efficacy and selectivity of treatments based on nanomaterials by combining chemo- and phototherapies, analyzing the correlation between surface chemistry, dimensionality, their combination with traditional drugs, and the application of laser pulses, something scarce in the scientific literature.

## 2. Methodology

In this study, the PRISMA 2020 (Preferred Reporting Items for Systematic Reviews and Meta-Analyses) methodology was used [25] to assess the studies related to the applications of GO and rGO against bone cancer between 2004 and 2022. In this sense, Elsevier Scopus was used as the primary database to comprehensively review the information from the citations and abstracts of scientific journals [26]. Scopus is considered one of the databases that houses the most relevant scientific information, given its vast diversity of publishers and journals [27]. In addition, it houses more than 25,100 titles from more than 5000 international publishers [26]. On the other hand, Scopus has several screening and bibliometric analysis functions that make it an attractive database; the most relevant are the name of the journal, type of document, year of publication, authors, and index (h-metric), among other options. In addition, it allows the downloading of the search in CSV format [28].

PubMed was also consulted as a secondary database to complement the search in Scopus [29]. The journal’s impact factor (IF) of the articles in the bibliometric analysis (supporting information) was obtained from the Journal Citation Report (JCR) 2022 and the Cite Score 2022 from Scopus. VOSviewer (www.vosviewer.com; Van Eck and Waltman, 2009–2022, version 1.6.18, Leiden University, Leiden, The Netherlands) is a free access software used to create network maps of the institutions, countries, keywords, and citations per article [30]. We accessed VOSviewer on 4 June 2023.

### Design of the Search Strategy and Eligibility Criteria for the Systematic Review

**Figure 1:**
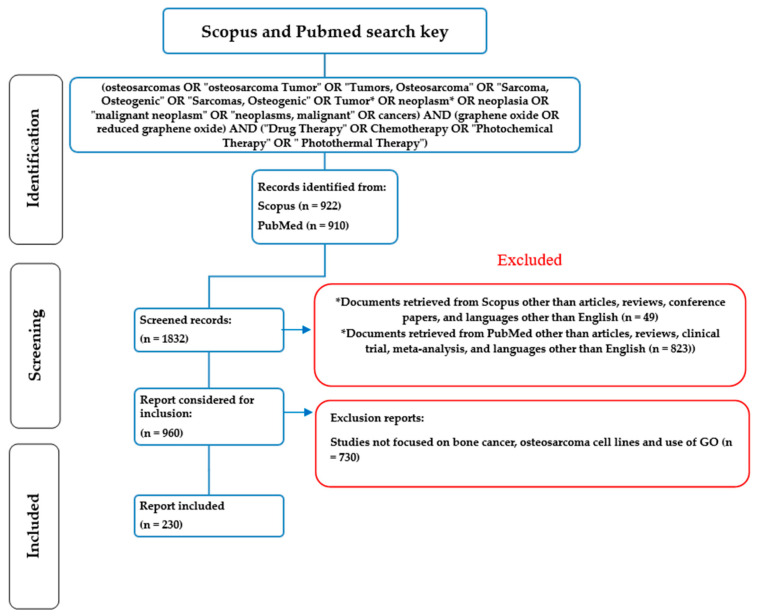
A flowchart of the PRISMA methodology used in the systematic review with bibliometric analysis [25].

The publications related to the study of GO and rGO and their application to the treatment of bone cancer were obtained using the following search key in Scopus: TS = ((osteosarcomas OR “osteosarcoma Tumor” OR “Tumors, Osteosarcoma” OR “Sarcoma, Osteogenic” OR “Sarcomas, Osteogenic” OR Tumor* OR neoplasm* OR neoplasia OR “malignant neoplasm” OR “neoplasms, malignant” OR cancers) AND (graphene oxide OR reduced graphene oxide) AND (“Drug Therapy” OR Chemotherapy OR “Photochemical Therapy” OR “Photothermal Therapy”)). This search yielded 922 (retrieved from Scopus on 4 June 2023) articles filtered by titles, abstracts, and keywords (Figure 1). With this same search key, 910 articles were retrieved in PubMed, a total result of 1832. Subsequently, the search was refined, limiting the type of document to “article,” “review,” or “conference paper,” and the English language, which allowed the exclusion of 49 documents in Scopus and 823 documents in PubMed, which generated 960 documents. These results removed 730 papers not addressing bone cancer, osteosarcoma cell lines, or use of GO, yielding only 230 articles for this study.

## 3. Results and Discussion

### 3.1. Review of Bone Cancer and the Use of GO for Biomedical Applications

#### 3.1.1. Osteosarcoma

**Figure 2:**
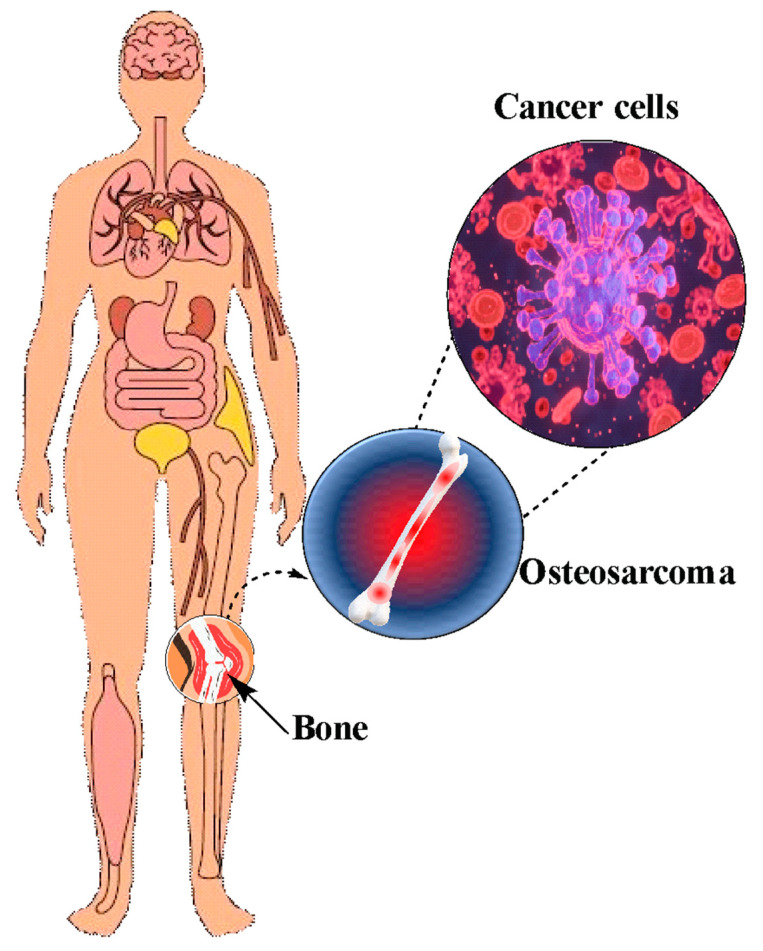
Osteosarcoma diagram in human bones. ©[Barba] via https://www.canva.com/ (accessed on 27 November 2023).

Osteosarcoma is a malignant neoplasm in which neoplastic cells generate bone, the most common primary sarcoma in bone (Figure 2). It is considered preliminary when the underlying bone is normal and secondary when there are significant alterations in the structure of the affected bone [31]. It is estimated that around 70% of the people who develop this disease are between the ages of 10 and 16, and about 30% of the cases in patients who are older than 40 years [32]. This disease can manifest in any anatomical location, with an incidence of 30% in the distal femur, 15% in the proximal tibia, and 15% in the proximal humerus [33].

Bone sarcomas are diagnosed at a rate of approximately 3.4 cases per million of the population worldwide [34]. In this sense, about half of the reported cases are associated with young people, which makes this a disease with premature death. In addition, several risk factors associated with this disease have been identified, such as radiotherapy and chemotherapy treatments [35]. It has been shown that these treatments have a mutagenic effect on the TP53 and Rb genes (tumor suppressor genes that are also involved in the regulation of the cell cycle), which is associated with the uncontrolled growth of osteocytes that produce immature bone, generating a palpable mass or bone tumor [36,37].

In this context, mesenchymal stem cells (MSCs) in the bone microenvironment are important factors promoting osteosarcoma metastasis [38]. For example, the alteration or absence of the TP53 and Rb genes, aneuploidization, and genomic deficiency of *P16/Cdkn2a* are frequent causes of the transition of MSCs to osteosarcoma cells [39,40]. In addition, there are various alterations or aberrant expressions of genes that induce the development of osteosarcoma. Genes such as *AP-1*, *c-myc*, *c-fos*, *MMP*, *TWIST*, and *IGF-1*, among others, have been reported as such [41,42,43,44]. Some incidences, risk factors, and the importance of the bone microenvironment in cancer progression have been addressed, as well as different risk factors in the treatment of osteosarcoma.

#### 3.1.2. Conventional Treatments and the Application of GO in the Treatment of Bone Cancer

Conventional treatments include surgical removal of the tumor at an advanced stage of the disease to save the affected limb. However, this treatment is inefficient given that there are high probabilities of disease recurrence through bone metastasis, with tumor metastasis being the main problem for tumor therapy [45,46]. Fortunately, technological and research advances in recent decades have allowed for the development of functionalized biomaterials that reduce the risk of amputation and death [47].

On the other hand, radiotherapy is a sophisticated, valuable technique in patients treated with multidrug chemotherapy who cannot undergo complete resection or are at an early stage of the disease [48]. However, its use is increasingly limited due to its side effects. For example, targeted radiotherapy with samarium-153-ethylenediamotetramethylene phosphonate has been used in extreme cases, even though its use is not well defined, and the secondary effects cause severe damage to non-target cells, causing malformations or alterations in osteoblasts [49,50,51]. In this sense, it is worrying that most conventional treatments for osteosarcoma consist of preparative chemotherapies.

Conventional therapeutic approaches for treating this disease are heterogeneous and based on the state and location of the tumors; these approaches are often inefficient in disease detection and treatment [8]. Unfortunately, these therapeutic approaches have not changed significantly and have been used for over 30 years. However, novel approaches based on non-invasive methods, such as liquid biopsy and the development of molecular biomarkers, have been reported, allowing for early disease identification to facilitate medical treatment [52].

On the other hand, during the last decade, unconventional methods based on nanomaterials for treating bone cancer have developed significantly, where GO has been an essential participant in various fields of study. GO is a two-dimensional nanomaterial with a thickness of approximately 0.6 nm, derived from graphene and relatively dispersible in water; it has hydroxyl (-OH), epoxy (-O-), carbonyls (-CO), carboxyl (-COOH), and carbon-carbon sp^2^ and sp^3^ with hydrophobic domains [53]. Several experimental investigations have demonstrated the usefulness of GO as a substrate for drug adsorption [54,55]. Likewise, several in silico studies based on the Density Functional Theory (DFT) have demonstrated the absorption mechanisms of drugs such as cytarabine [56], 5-fluorouracil [57], and thioguanine [58], among others, which is essential given that the surface area of this nanomaterial and its molecular topology facilitates the adsorption of various drugs that are released in a controlled manner in response to pH changes. Similarly, the design of CePO_4_ nanorods modified with GO and chitosan (CS) has been successfully used to treat bone metastasis through photothermal therapy to inhibit tumor growth [59]. Additionally, the synergistic effect of the conjugation of GO and zoledronic acid (GO-ZOL, a nanostructured material) has shown high efficiency for the treatment of osteosarcoma through the mineralization of cancer cells, as studied by Boran et al., who reported the efficiency of GO-ZOL nanostructures as promising drug complexes for the treatment of bone cancer due to their stability/solubility in physiological environments, non-toxicity, and broad surface area [60].

#### 3.1.3. GO and rGO Synthesis

**Figure 3:**
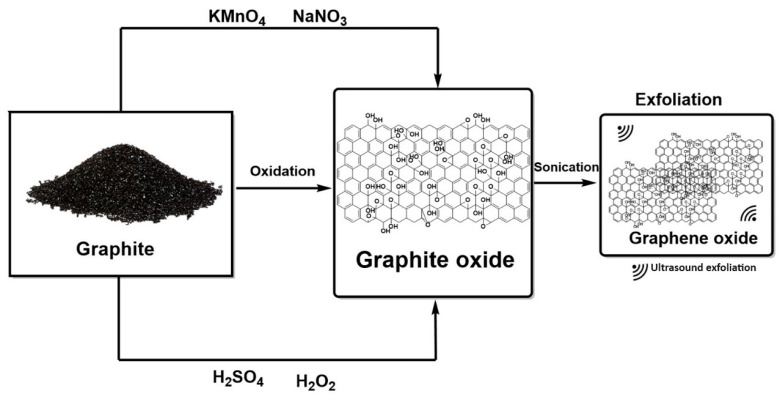
Hummer–Offeman method of GO synthesis.

The first synthesis of GO was seen in 1859 when the British Benjamin Brodie proposed it; this synthesis was based on the oxidation of graphite by potassium chloride (KClO_3_) and concentrated nitric acid (HNO_3_) [61]. Brodie determined that a compound formed of carbon, oxygen, and hydrogen, which he called graphic acid, was obtained with this process. However, this method was modified by the German chemist Staudenmaier, who slowly added concentrated sulfuric acid (H_2_SO_4_) to Brodie’s formulation. This method increased the graphite’s oxidation rate [62]. Likewise, the chemists Offeman and Hummer modified this formulation using a mixture of sodium nitrate, sulfuric acid, and potassium permanganate at 45 °C, stirring for two hours to obtain a gray paste [63]. This paste is diluted in water with hydrogen peroxide to increase the degree of oxidation and eliminate suspended manganese. Until now, this method, known as the “Hummer-Offeman method” (Figure 3), is the most accepted method for synthesizing GO. Thus, any technique modifying the Hummer–Offeman method is known as a modified Hummer [64]. For example, a modified Hummer method replaces sodium nitrate with phosphoric acid and sulfuric acid (H_2_SO_4_/H_3_PO_4_ (9:1)) and increases potassium permanganate. This modification reduces the production of toxic gases such as N_2_O_4_, NO_2,_ and ClO_2_ and increases the oxidation degree [65]. 

On the other hand, chemical reduction with hydrazine hydrate leads to the synthesis of rGO [66]. However, it is possible to use various reduction methods (including natural chemical compounds such as ascorbic acid, green tea extract, and curcumin) to identify the one with the best reducing capacity but the most negligible environmental impact to prepare rGO.

#### 3.1.4. Action Mechanisms

GO has unique properties that make it a promising candidate for bone cancer treatment. The mechanisms through which GO’s therapeutic effects diminish bone cancer are attributed to its ability to functionalize with specific targeted ligands, such as antibodies or peptides, that selectively bind to cancer cells [67]. This targeted approach allows for the delivery of anti-cancer drugs directly to the tumor site, minimizing side effects and reducing systemic toxicity.

The ability of graphene oxide to generate reactive oxygen species when it penetrates the cell induces oxidative stress, causing damage to molecules, mitochondrial malfunction (releasing cytochrome c), and affecting organic structures, among other effects. These events lead to DNA or RNA damage, causing alterations in cell signaling pathways and lipid peroxidation and the destruction of membrane structures, resulting in cell death by apoptosis or necrosis [68]. 

Upon contact with the cell, graphene oxide can pass through cell membranes and interact with the lipid tails of these membranes, leading to the extraction of hydrophobic cholesterol and generating pores that cause damage and affect its cellular function [69]. In addition, graphene oxide has been observed to adsorb nucleotides to protect them from the nuclease enzymes responsible for breaking down nucleic acids.

Additionally, GO has proven to be a good candidate for photothermal therapy due to its excellent photothermal properties, which means that it can convert near-infrared light into heat [70]. When GO is exposed to near-infrared light, it generates localized hyperthermia, destroying cancer cells. This approach can selectively target tumor cells without affecting surrounding healthy tissues [71]. There are several types of hyperthermia depending on the temperature range: mild (physiological temperature 43 °C), moderate (temperature range above the soft threshold, between 43 and 50 °C), and ablative hyperthermia (with a temperature range of 50 and 55 °C); the latter causes serious effects [72,73,74]. Some studies use electromagnetic radiation to irradiate photosensitive nanomaterials to release heat, as in the case of photothermal therapies, which use wavelengths of 650–950 nm (NIR-I) and 1000–1350 nm (NIR-IIa). At these wavelength ranges, the energy has a maximum penetration into the affected tissues [75].

In addition, GO exhibits optical solid and magnetic properties, making it suitable for various imaging modalities, including fluorescence imaging and MRI [76]. These imaging techniques can provide real-time visualization of the tumor and aid in accurate diagnosis, treatment planning, and the monitoring of therapeutic response. GO has been shown to interact with the cell signaling pathways involved in cancer progression and metastasis. It can modulate the expression of genes and proteins related to cell proliferation, apoptosis, angiogenesis, and the immune system [77]. These interactions can inhibit tumor growth and improve the efficacy of other treatment modalities.

#### 3.1.5. GO’s Anti-Cancer Applications

GO and rGO are two-dimensional nanomaterials derived from graphene (the oxidized and reduced form are derived by various methods, such as the Hummers method), which have hydroxyl, carbonyl, epoxy, and carboxylic groups [78,79]. This material presents attractive properties such as biocompatibility and physiological stability [80], biodegradability and mechanical resistance [81], good capacity for drug release [82], and a good surface area that facilitates the functionalization of the material [83,84].

Functionalizing these nanomaterials has made it possible to optimize the specificity of conventional chemotherapeutic methods, improving their specificity for cancer cells and improving the controlled release of chemotherapeutic drugs at the tumor site. In addition, it has significantly reduced the side effects of chemotherapy [85]. On the other hand, using bone cement in treating osteosarcomas is common; however, this bone cement does not significantly inhibit cancer cells. Functionalized bone cement has been developed to overcome the inhibitory effects of cancer cells by coprecipitating GO and tricalcium silicate nanoparticles. This functionalization improved photothermal performance for the minimally invasive therapy of bone tumors.

Furthermore, functionalized cement promotes cell proliferation and the alkaline phosphate activity of MC3T3-E1, making it a promising material for in vivo applications [86]. Likewise, the in vitro application of hydrogels based on nano-hydroxyapatite (nHA) and rGO (nHA-rGO) has been successful in the design of photothermal therapies for the treatment of bone cancer; this hydrogel showed an efficiency of up to 90% for the inhibition of osteosarcoma cells (MG-63) [87]. The design of functional materials from the functionalization of GO, such as the synthesis of GO nanoribbons, is attractive for biomedical applications due to the ease of incorporating functional groups that confer properties of interest to the researcher, which are valuable for regenerative medicine [88].

##### In Vitro Studies

In recent decades, the application of carbon-based nanomaterials such as GO and rGO has had rapid growth in science and technology. For example, several nanocomposites based on nanosheets of carboxylated GO (GO-COOH) and doped with Zinc oxide nanoparticles (GO-COOH/ZnO) have been obtained through the carboxylation of GO and the nucleation of ZnO in the nanosheets; this nanomaterial has shown excellent osteogenic activity through its in vitro evaluation [89]. These results are interesting for tissue engineering because they also significantly stimulate osteocalcin production (which prevents locoregional relapse after surgical treatment) and mineralize the extracellular matrix of MG-63 osteoblasts.

Similarly, scaffolds from the graphene family have been used in regenerative medicine and bone tissue engineering due to the desirable properties of these materials, such as improved mechanical properties, surface area, and a variety of functional groups [90]. This has allowed for the study of in vitro properties of graphene-consistent scaffolds and their osteogenic properties that will enable the evaluation of their in vivo performance [91].

In vitro studies make it possible to evaluate the cytotoxicity and biocompatibility of graphene oxide, along with the toxicity that it may present, depending on its inherent characteristics and external factors. The response to these nanomaterials will depend on its number of layers, hydrophilicity, purity, and surface chemistry [92]. 

Variations in GO concentration in different cell types over specific periods can be examined. Niu et al. [93] investigated a functionalized graphene-dendrimer system for the delivery of doxorubicin (DOX) and melatonin (MLT) over Saos-2, MG-63, and hBM-MSC cell lines. An in vitro cytotoxicity analysis showed an 85–100% viability at treated doses from 10 to 1280 μg/mL, indicating low cytotoxicity and excellent biocompatibility. Similarly, Saravanabhavan et al. [94] demonstrated that graphene oxide functionalized with chitosan nanoparticles targeting Saos-2 and MG-63 osteosarcoma cells induced cytotoxicity. The GO/CS compound was less toxic, with a cell viability greater than 0.55 ± 0.75 compared to the control (viability of 0.63 ± 0.65) after 48 h. In addition, the viability of 0.4 ± 0.43 and 0.49 ± 0.53 was verified for Saos-2 and MG-63, respectively, at the highest concentration of 100 microns, with Saos-2 cells being more susceptible to treatment as they have a lower viability. These in vitro studies enable the analysis of the inherent cytotoxicity of materials, contributing to a more robust understanding for subsequent in vivo testing.

##### In Vivo Studies

Given the rapidly growing application of graphene-based nanomaterials, it is essential to be rigorous in assessing the biocompatibility of GO nanomaterials under in vivo applications since several in vitro and in vivo studies have shown some degree of reliability but with non-target cell toxicity [95]. For example, it has been shown through in vivo studies that GO can induce hemolysis in cells due to electrostatic-like interactions between the GO surface and the lipid bilayer of non-target cells [96]. However, this effect of GO is essential for developing therapies against osteosarcoma cells resistant to conventional drugs. 

The in vivo fate of graphene and GO is influenced by several factors, such as their interaction with biological systems, biodistribution, toxicity, excretion, route of administration, and nanomaterial size. The in vivo system allows for the evaluation of the toxicity that graphene-based nanomaterials can induce, the interaction between the cell and graphene oxide via histological examinations, and the inflammation of cells after oral, intravenous, skin injection, and intraperitoneal administration [97]. In this regard, Li et al. established the stable and non-covalent combination of trastuzumab (ART) with graphene oxide for the generation of a TRA/GO complex on cells in osteosarcoma (OS) [98]. The TRA/GO complex showed enhanced HER2-binding activity, increasing its ability to kill OS cells and inducing oxidative stress. Intravenous administration of the TRA/GO complex (5/1 mg/kg) twice weekly eradicated an OS xenograft in immunodeficient mice, halting tumor growth after treatment and thereby increasing survival. Histological and pathological analyses did not identify the presence of any tumors in the lung section of ART/GO-treated mice compared to GO- or ART-treated mice.

In this sense, synergistic photodynamic/photothermal therapy, or targeted phototherapy, is a promising strategy for treating bone tumors resistant to traditional medicines [99]. Recently, Zeng et al. studied GO nanocomposites targeting the mitochondria of MG-63 cancer cells for synergistic photothermal therapy. This study consisted of the conjugation of triphenylphosphonium (PPG) (4-carboxymethyl) phosphonium bromide (TPP) conjugated with indocyanine green (ICG)-loaded pegylated GO nanosheets (TPP-PPG@ICG), which promoted the accumulation of mitochondria after administration of the material; at this point, near-infrared irradiation led to the inhibition of ATP and mitochondrial function, causing cell death in target cells [22].

Additionally, the size of the two-dimensional GO and rGO nanostructures (between 430 and 780 nm) is a determining factor in their toxicity against osteosarcoma cell lines (Saos-2 and MG-63), since they can penetrate cells and release drugs in a controlled and selective manner. In this sense, these nanomaterials have been used successfully as carrier vehicles for drugs such as doxorubicin (DOX) against osteosarcoma in the Saos-2 and MG-63 cell lines [93]. Table 1 summarizes some notable applications of these nanomaterials in various cell lines associated with osteosarcoma (Saos-2 and MG-63). In addition, the type of study, the method of analysis used, and the results of each study are shown in tags. In vivo studies of the materials will depend on different factors, such as the physiological environment in which they are tested, the routes of administration, and the host’s immune system, among others. In vivo studies are equally as important as in vitro studies, allowing for a greater understanding of the host’s biological or metabolic systems.

### 3.2. Photodynamic Therapy 

**Figure 4:**
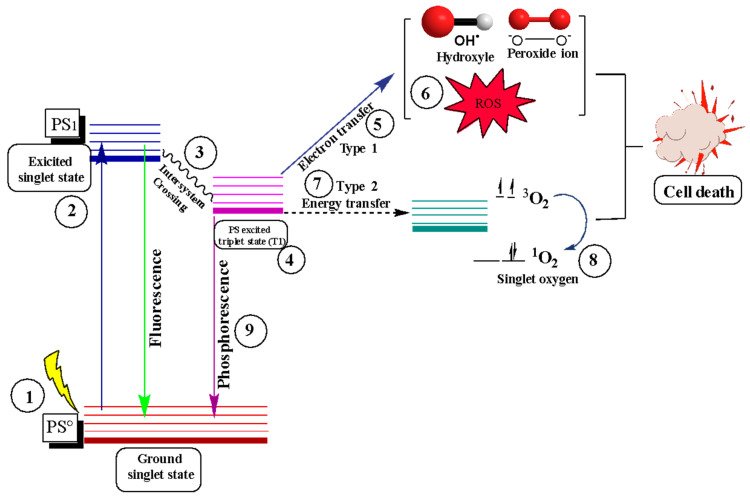
Diagram of photoreactions of type 1; transfer of an electron (5), free radical production (6), and type 2; transfer of energy directly to the oxygen molecule (7), formation of singlet oxygen (8). Subsequently, cell death by photodynamic therapy is seen. Adapted from [106,109].

Photodynamic therapy is non-invasive in different non-oncological diseases, specific cancers, dermatology, and urology [105]. Photodynamic therapy consists of the use of photosensitizers (PS) that are irradiated by a light or a beam of light with a specific wavelength once it enters the cell (1), generating a singlet excitation state (2); the high energy produces fluorescence, and the other part of the energy, due to the crossing of intersystem (3), is maintained in another state called the excited triplet state (4), allowing the PS to interact with various molecules in the cell [106]. In the excited triplet state, two types of photoreactions can occur, one in which an exchange of an electron occurs between the PS and the substrate of importance (5) (Figure 4). The reaction of oxygen molecules with this electron generates a form of oxygen called oxygen radicals and reactive oxygen species (ROS), such as peroxides and hydroxyl radicals (6). The damage is caused by oxidative stress and, therefore, the breaking of bonds [107]. 

On the other hand, in the second photoreaction (7), the interaction occurs directly with the oxygen molecules but at a basic triplet level. It produces singlet oxygen and the reactive oxygen species (8). It should be noted that, in the triplet state, the PS can return to its basal state by emitting phosphorescence (9). However, this type of energy is not essential because it requires that the energy received by the photosensitizer allows for interaction with the biomolecules of the cell. The excited triplet state is more viable than the singlet state due to its stability. In the singlet state, the duration of the reaction is in nanoseconds, without allowing for the interaction between biomolecules and the photosensitizer, while in the triplet state, the duration of the response is in microseconds, allowing the molecular oxygen to receive the energy of the photosensitizer to generate the interaction [108]. 

#### Photodynamic Therapy as a Cancer Treatment 

Photodynamic therapy (PDT) is a therapy used to kill cancer cells, a treatment that consists of the use of photosensitizing drugs that, when activated with a specific light or beam of light, interact with some molecules of the cell; generally, these molecules produce singlet oxygen and reactive oxygen species (ROS). The light source for drug irradiation comes from a laser, a light-emitting diode (LED), or another source that allows for activation [110]. Generally, the specific wavelength range for tissues is 600–1200 nm; however, a range of 600–800 nm is used for photosensitizers, since, at a longer wavelength, singlet oxygen would not be produced, and less light scattering is achieved in the tissues, with a better absorption of photons in the region of interest [107]. 

This contrasts with what Mfouo-Tynga proposes, who argues, through their studies, that using two-photon photodynamic therapy can reach tumors with an optical window greater than 800 nm, thus obtaining a greater penetration into deeper tissues [106]. The light source will depend on the photosensitizer’s absorption range, the tumor type, and the tissue’s optical window [108,111]. 

Photodynamic therapy affects tumor-associated immune cells, leading to apoptosis or necrosis [112,113]. When interacting with molecules in the environment, the photosensitizer, either via energy transfer or an electron, produces singlet oxygen and ROS, generating oxidative stress damage in proteins and nitrogenous bases in nucleotides and lipids [114]. Mitochondria are susceptible to the oxidative stress caused by reactive oxygen species, which would affect their functioning and ATP production [113,115]. For example, a study of murine B16-F10 melanoma cells, by applying methylene blue as a photosensitizing agent, found that when methylene blue is activated by visible light variation and the drug, apoptosis and cell necrosis occur. Cell necrosis occurred immediately after the application of photodynamic therapy with methylene blue as a photosensitizer, thus altering the cell membrane with a minimum energy of 16.8 J. Apoptotic death was reported to have occurred between 6 and 24 h, producing changes in the nucleus and generating chromatin fragmentation. The study reported 30% to 40% cell apoptosis with a methylene blue concentration of 2 μg/mL and an energy variation of 100.8 J [116].

On the other hand, it was studied how the combination of carboplatin and photodynamic therapy with 9-hydroxypheophorbide α (chlorophyll-derived photosensitizer) improved the apoptotic process in AMC-HN-3 laryngeal cancer cells. Cytotoxicity was measured by the drug interaction coefficient (CDI) of the combination of carboplatin and photodynamic therapy with a concentration of 0.04 mg/mL using different concentrations of 9-hydroxypheophoric α, obtaining a value of 0.28 CDI, indicating a synergistic effect in the combination. When 9-hydroxypheophorbide α is stored in the mitochondria and endoplasmic reticulum, apoptosis occurs. In the mitochondria, stress is generated in the mitochondrial matrix, activating the enzyme caspase 9 at the beginning of the process and ending with caspase 3, inducing apoptosis [117].

An essential aspect of photodynamic therapy is that it is non-invasive, and the photosensitizers used are non-toxic. Many studies have reported using photodynamic therapy in different types of cancer, such as breast cancer [118], skin cancer [119], esophageal cancer [120], and bone cancer, among others [121]. Photodynamic therapy is a widely used alternative in bone cancer that invades bone tissue, such as osteosarcoma, which causes immobilization and can metastasize to the lungs [122]. 

**Figure 5:**
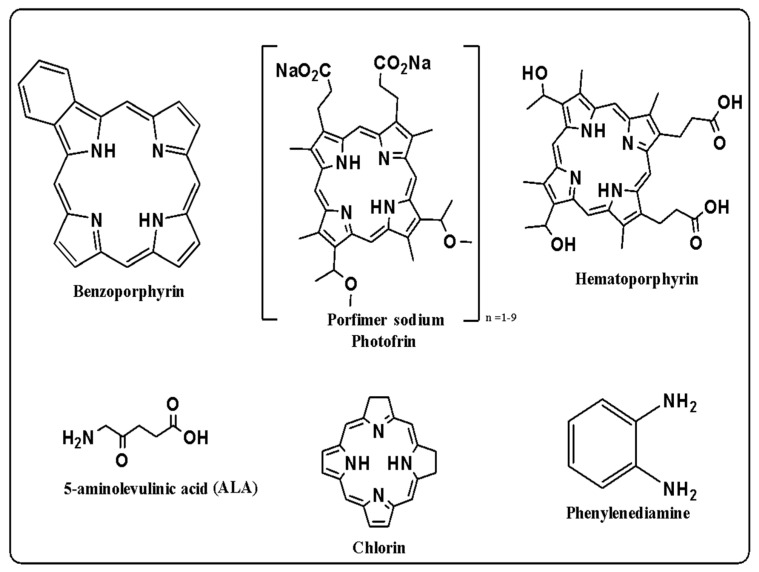
Chemical structures of the most common photosensitizers used in photodynamic therapy.

In treating different types of cancer, other photosensitizers have been used for tumors, which can be classified into first-generation, second-generation, and third-generation. First-generation photosensitizers (Figure 5) include porphyrins and their derivatives. Hematoporphyrin was one of the first photosensitizers used on a large scale against cancer. Photofrin, the first photosensitizing drug approved by the Food and Drug Administration (FDA), was then obtained. However, porphyrins and their derivatives exhibit low chemical purity, are less selective, and exhibit skin hypersensitivity [123]. Second-generation photosensitizers are derived from hematoporfins and synthetic compounds such as chlorines, phthalocyanes, benzoporphyrins, thiapurines, and 5-aminolevulinic acid, among others [124]. Each of these drugs belonging to the second generation of photosensitizers has high tissue penetration, fewer side effects, as well as high absorption at near-infrared wavelengths, and a high degree of singlet oxygen production; however, there are limitations in their penetration into deeper tissues [105,106]. Subsequently, third-generation photosensitizers were developed, which improve the administration of photosensitizers and their selectivity through the combination or conjugation of second-generation photosensitizers with carbohydrates, amino acids, peptides, and their combinations with lipoproteins, and nanoparticles, among others [107,124]. 

Recently, the effect of photodynamic therapy with 5-aminolevulinic acid in combination with cis-platinum on OVCAR cells in ovarian cancer has been studied, demonstrating that combining photodynamic therapy with a second-generation photosensitizer and cis-platinum inhibits cell proliferation and induces cell death by apoptosis [125].

Photodynamic therapy has been widely studied in osteosarcomas due to the drug resistance of this type of cancer; combinations of natural photosensitizers with silica nanoparticles to obtain a better bioavailability and solubility of the photosensitizer have been reported [126]. For example, Ghaseb et al. used an extract from the flowers and aerial parts of chicory grass (*Cichorium pumilum*) that was loaded with silicon nanoparticles to evaluate the encapsulation effect on the efficacy of chicory as a photosensitizing agent in photodynamic therapy, with good encapsulation efficiency, increasing its capacity to absorb light and generate reactive oxygen species (ROS) [127]. Yu et al. proposed a therapeutic approach to treating osteosarcoma that involves combining surgical tumor resection with photodynamic therapy activated with bovine serum albumin and zinc phthalocyanine [128]. The results highlight the significant impact of photodynamic therapy on regulating the PD-L1 programmed death ligand in tumor cells. This phenomenon inhibits the activity of cytotoxic T lymphocytes and compromises the immune response against the tumor. In addition, an effective inhibition of autophagy was observed, contributing to a more efficient immune response and suppressing osteosarcoma in both in vivo and in vitro studies. This approach suggests a promising application for preventing tumor metastasis. On the other hand, Huang et al. surveyed the ability of graphene oxide nanoparticles conjugated with polyethylene glycol, along with folic acid (FA) and the green indocyanine photosensitizer (ICG) (PEG-GO-FA/ICG) to suppress the inhibitor MTH1, which plays a crucial role in repairing oxidative DNA damage [129]. The combination of photodynamic therapy and chemotherapy inhibited the proliferation and migration of osteosarcoma cells. In summary, combining photodynamic therapy with multiple therapeutic approaches reveals significant potential to treat osteosarcoma. Table 2 provides additional information on photodynamic therapy systems’ applications against osteosarcoma.

### 3.3. Photothermal Therapy

**Figure 6:**
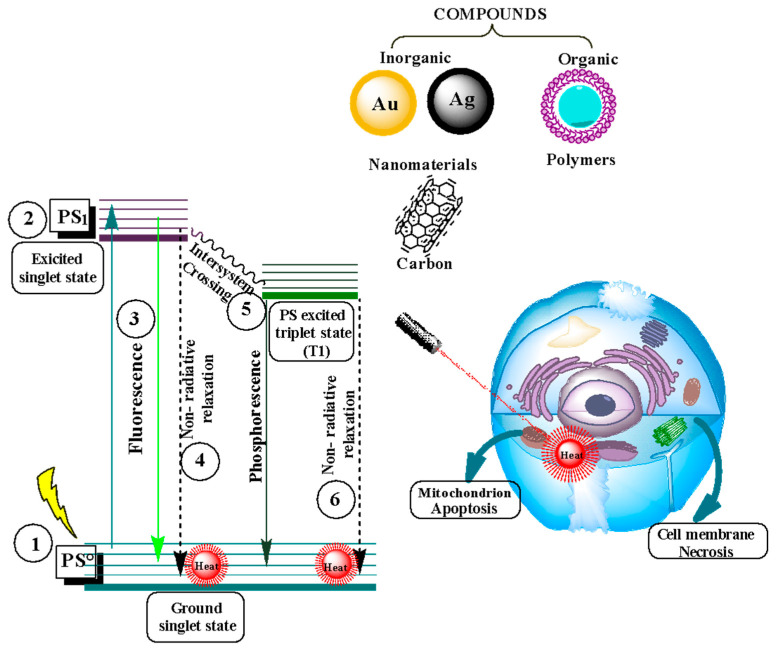
Outline of photothermal therapy and the processes that can occur after excitation from the base level (1) to the excited level (2). Fluorescence (3), non-radioactive relaxation (4 and 6), and intersystem crossover (5).

Photothermal therapy (PTT) is based on irradiation using light of a single wavelength in the near-infrared (NIR) range, ranging from 750 to 1000 nm. This light is absorbed by photosensitive agents that convert it into heat, generating a photothermal effect that produces a controlled temperature increase in a localized manner [123,132]. Figure 6 shows a photothermal diagram with a photosensitizer (PS) that is irradiated using a beam of light of a specific wavelength (1), thus generating a state of excitation from PS° to the PS1 state (2). During this base-to-excited level excitation, several processes can occur. The first is radioactive transition, where a photon is emitted as fluorescence (3). Then, non-radioactive relaxation converts the dissipated energy into heat (4) [133]. Finally, a triplet state is generated due to the intersystem crossing (5), where interaction occurs with the oxygen molecules found in the medium (a process used in photodynamic therapy). In turn, these triplet state molecules can undergo non-radioactive relaxation (6) [108].

Photothermal therapy uses the photothermal lethal effect produced by the photothermal agent (PTA) when converting absorbed light into heat. Photothermal agents can consist of organic and inorganic compounds. The most commonly used inorganic compounds are noble metals such as gold (Au) [134], silver (Ag) [135], palladium (Pd) [136], and platinum (Pt) [137]; carbon-based compounds can also be used, such as graphene [138], graphene oxide, and carbon dots [139]. Organic compound-based photothermal therapy uses polymer nanoparticles [140] and organic dyes [141], among other materials [142]. The effectiveness of the photothermal treatment will depend on the ability of the PTAs to convert absorbed light into heat. These characteristics of PTAs can be enhanced by combining materials that increase the penetration and tissue specificity and those that decrease potential side effects [143].

Photothermal therapy is used to target localized tumors [144], treatments in ophthalmological diseases [145], bacterial and microbial infection [146], dermatological diseases [147], tissue repair [148], and the release of chemotherapeutic drugs [149] in a highly selective and minimally invasive manner, with short treatment times [150]. Ting et al. demonstrated that hybrid polymer nanoparticles composed of methoxy polyethylene glycol-benzoic-imino-1-octadecanamine (mPEG-b-C18), hydrophobic poly(lactic-co-glycolic acid) (PLGA), and polyethylene glycol tocopheryl succinate (TPGS), called PBCTPN, and carrying indocyanine green (ICG) (a photothermal agent of amphiphilic nature approved by the FDA), improved the aggregation, photostability, and release of ICG. This release occurred when the pH was lowered from 7.4 to 5.5, which generated acid hydrolysis with the benzoic imine of mPEG-b-C18, allowing a separation of the mPEG and, subsequently, the release of ICG. In addition, an appreciable increase in stability and efficiency was observed, attributed to the spherical and well-dispersed shape of the nanoparticles [151]. Finally, PBCTPNs were compared with PLGA nanoparticles (PN) and mPEG-C18/TPGS/PLGA (PCTPN) nanoparticles, demonstrating that ICG fluorescence was markedly higher in the cytoplasm of MCF-7 cells treated with PBCTPN and PCTPN compared to cells incubated in ICG-free PN and PN loaded with ICG. The PEG coating on the surface of the nanoparticles (PBCTPN and PCTPN) partially protects the ICG molecules’ negative charges, reducing the electrostatic repulsion forces between the nanoparticles and cell membranes that also possess negative charges. Therefore, ICG and PEG were more efficient at entering the cytoplasm of cancer cells. The ability of various photothermal agents to convert light into thermal energy, combined with the irradiation of light at the appropriate wavelength, makes photothermal therapy one of the non-invasive methods for cancer treatment.

#### Photothermal Therapy as a Cancer Treatment

Photothermal therapy has been used against cancer as it is a less invasive therapy with high specificity and selectivity [152]. This is due to photothermal agents that, by converting light into heat, increase the temperature of the tumor without affecting healthy tissue. This is also because external irradiation allows for temperature control and greater precision [153]. Photothermal therapy is known to be efficient when combined with other modalities, such as chemotherapy [149] and the use of nanostructures [154], improving its therapeutic characteristics, such as anticancer efficacy, and minimizing limitations due to drug resistance [155]. For example, visible irradiation with gold nanoparticles has been used to enhance the cytotoxic effect of doxorubicin (DOX) in breast cancer, increasing its therapeutic efficacy by 77% [154].

Cancer can develop resistance to different treatments and drugs that target different molecular pathways [156]. Drug resistance can occur intrinsically, involving membrane transporter proteins, ATP cassette binding transporters (ABC), and solute transporters in a way that alters drug transport, interfering with the cellular absorption of anticancer agents [157,158]. For example, glycoprotein P, an ABC protein, is one of the main factors responsible for drug resistance because it expels drugs through a membrane pore, preventing them from concentrating inside the cell [156,159].

On the other hand, in acquired resistance, promising results are initially obtained during chemotherapy; however, due to the multidrug resistance (MDR) generated by genetic mutation activities during treatment, poor outcomes begin to develop during treatment [156,157].

Generally, the drugs used in cancer have low bioavailability, low permeability, and poor absorption, causing high doses of the drug to be required and adverse side effects to health [160,161]. However, thermal therapy mediated by the combination of nanoparticles, organic compounds, and inorganic compounds can incorporate stability and greater efficiency, introducing the conversion of light into heat and reducing the high doses of the drug [162]. The advantage of using nanomaterials lies in their smaller size, quick entering of tumor cells, which generally promotes the proliferation of abnormal blood vessels, increasing their accumulation in the tumor, and their more excellent vascular permeability, creating a better therapeutic burden in response to the tumor environment [163]. Graphene oxide (GO), when functionalized with other compounds, increases its hydrophilicity and biocompatibility, allowing it to penetrate the tumor easily and decrease toxicity [164]. Graphene oxide-derived compounds are used as photothermal agents due to their high capacity to conduct electricity and heat and increased ability to absorb light, making them a potential material for use in combined photothermal–photochemical therapies [165] (Table 3).

Combining graphene oxide and magnetite functionalized with hydroxypropyl cellulose and paclitaxel (an antineoplastic agent used in breast and ovarian cancer) improved their biocompatibility. Their better compatibility is due to the favorable interaction between GO, hydroxypropyl cellulose, and magnetite due to hydrogen bonds, allowing greater efficiency in localized heat release and the drug. This characteristic was attributed to the localized heat and protonation of NH groups present in paclitaxel, which increased their release, achieving greater efficiency in destroying tumor cells [166].

On the other hand, the irradiation of graphene oxide (GO), reduced graphene oxide (rGO), and the photosensitizers IR-780, IR-780-rGO, and IR-780-rGO-HA, with a light beam of 808 nm for 3 min, maintaining a fixed concentration of 0.4 mg/mL and a variable amount of IR-780, allowed the obtaining of maximum temperatures of 41.3, 54.4, 37.5, and 58.6 °C for GO, rGO, IR-780, and IR-780-rGO, respectively. This indicated a more significant photothermal effect on graphene oxide and reduced graphene oxide; however, rGO possesses a more significant photothermal conversion effect, and covalently binding with IR-780 increased the ROS production for photodynamic therapy. Hyaluronic acid was used as a coating for IR-rGO and conferred an increased intracellular uptake by cancer cells, blocking CD44 receptors on the cell surface of U87. Interestingly, the side effects that doxorubicin can generate were minimized using the nanocarrier IR-780-rGO-HA. In addition, another great advantage of combination therapy is that it presented an increase in the release of DOX at an acidic pH to 67.3%, decreasing the use of high doses required for treatment [167,168].

Photothermal therapy, by selectively destroying cancer cells using NIR irradiation, is recognized as a non-invasive and preferable approach to osteosarcoma treatment. It can improve diagnostic efficiency when combined with other therapeutic modalities, such as chemotherapy, drugs, and nanoparticles, with appropriate physical and chemical properties. Ma et al. developed a multifunctional scaffold using temperature-controlled nanohydroxyapatite/graphene chitosan oxide (nHA/GO/CS) to target osteosarcoma cells efficiently [169]. The GO/CS and nHA/GO/CS scaffolds raised the tumor temperature to 49.5 °C and 49.9 °C, respectively, after 150 s of laser irradiation. It was observed that by keeping the temperature of the tumor at 48 °C, the surrounding tissue had a low temperature, which reduced damage to healthy tissues. In addition, a significant decrease in tumor volume was recorded in those treated with the scaffolds, while those without irradiation showed rapid growth. This highlights that NIR irradiation, being artificially controlled, reaches the tumor site, thus treating osteosarcoma without side effects compared to other techniques. Yang et al. developed a nanomaterial containing iron, SiO_2_ @PDA/Fe^3+^—FA (PDA: polydopamine; AF: folate) to address osteosarcoma exhibiting some insensitivity to chemotherapy [170]. It was demonstrated that this nanoparticle showed high efficiency in delivering doxorubicin and cisplatin, along with an efficient photothermal effect. Furthermore, the presence of iron ions facilitated Fenton-like reactions, leading to the generation of reactive oxygen species crucial for inducing apoptosis in osteosarcoma cells. Photothermal therapy increases their selectivity and therapeutic index, thus improving the treatment of cancers that resist multiple drugs [171].

**Table 3:** Different types of photothermal therapy systems (PTTs) in osteosarcoma applications.

PTT System	* PTA	Type of Study	Analytical Method of Osteosarcoma Anticancer Activity	Results	Ref.
Nanomaterial of SiO_2_ @PDA/Fe^3+^	PDA	In vivo In vitro	The photothermal effect of xenograft tumor models in naked mice. Nanomaterials injected into the tail at a 20 mg/kg dose. Temperature 42.5 °C ± 0.5 °C. The dose of cis-platinum for MNNG/HOS cells and 143B cells was set at 6 mg/kg by intraperitoneal injection. The cis-platinum dose for MNNG/HOS cells and 143B cells was carried out according to the IC50 dose. This dose was 3 μM.	The burden percentages of doxorubicin and cisplatin were 21.25% and 23.80%, respectively. The system saw a considerable increase in the photothermal conversion efficiency of nanomaterials by 57.63% and photothermal conversion at low temperatures of 42–43 °C.	[170]
Scaffolds with DOX-gelatin/SrCuSi_4_O_10_-β-TCP core/ shell filaments.	CS	In vitro	The photothermal performance of gelatin-TCP/SC scaffolds with DOX was evaluated. To evaluate the anticancer effects in vivo, hTCP/2SC-DOX was carried out in mice with MG-63 cells. To evaluate the cytocompatibility of hTCP/SC scaffolds, BMSC and HUVEC were cultured.	Hyperthermia increased the release of DOX caused by NIR-II by irradiating the HtCP/2SC-DOX scaffolds with a 1064 nm laser for five cycles of 5 and 10 min. A synergistic effect was achieved in the combination of photothermal therapy and chemotherapy.	[171]
Multifunctional nano-hydroxyapatite/graphene oxide/chitosan (nHA/GO/CS) scaffold	GO	In vitro	TEM characterization. For photothermal performance: 808 nm NIR. MC3T3-E1 and HOS cells were cultured at 30% nHA/GO (50 μg/mL). MC3T3-E1 at temperature 42 ± 0.5 °C. Western blot, RIPA y BCA CCK-8 was used to evaluate cell activity. For the in vivo study, naked mice were used with NIR lasers (0.6 W/cm^2^).	The 30% nHA in GO increased biocompatibility and the photothermal effect to eliminate HOS cells. Excellent in vitro performance of the nHA/GO/CS scaffolding and efficient operation with NIR for osteosarcoma cell removal and tissue regeneration were achieved.	[169]
Methotrexate-loaded polydopamine (pDA)-based ZIF-8-based nanoparticles (pDA/MTX@ZIF-8)	pDA	In vitro	For photothermal analysis, an 808 nm NIR laser was used. MMP change was examined using fluorescence imaging.	The in vitro study demonstrated excellent antitumor efficacy by inducing apoptosis in MG63 cells. In addition, pDA/MTX@ZIF-8 nanoparticles showed good biocompatibility and an exceptional ability to release methotrexate as a function of pH, with 93% and 94.5% release in 12 h and 3 days, respectively. pDA/MTX@ZIF-8 nanoparticles exhibited a synergistic chemo-photothermal effect for cancer therapy.	[172]
Indocyanine green-loaded membrane-coated silica nanoparticles of cancer cells (CM/SLN/ICG)	ICG	In vitro In vivo	Characterization with TEM scanning electron microscopy. Western Transfer Assay. Analysis of 143B cells by flow cytometry. To evaluate the photothermal effect, mice with 143B cells with CM/SLN/ICG were used and irradiated with a wavelength of 808 nm.	It was shown that the photothermal conversion efficiency of CM/SLN/ICG and ICG was 57.93% and 57.21%, respectively, indicating that ICG generates higher protected photothermal conversion. In addition, it was found that the release at pH 5.5 and 7.4 was 74.41% and 32.96%, respectively. It was found that the anticancer efficacy was superior in modified CM, CM/SLN/ICG, and could specifically target 143B cells, enhancing its promise as a drug manager in TTP.	[173]
UiO-66 nanoparticles, polydopamine-coated with perfluorotributylamine/thyrazamine (TPZ/PFA@UiO66@PDA)	PDA	In vitro In vivo	Cell cultures of 143B were used for cell viability live/dead assay. 808 nm laser, flow cytometry for in vitro hypoxia and apoptosis analysis. For in vivo assay mice were injected intratumorally with 143B.	Tumor cell destruction was achieved by NIR irradiation and effective synergy between hypoxia-activated bioreducing prodrug therapy and TTP. The photothermal effect in vivo and in vitro at a temperature of 60.27 ± 3.02 showed that tumor size was significantly reduced, demonstrating an excellent antitumor capacity of TPZ/PFA@UiO66@PDA nanoparticles.	[174]

Abbreviations: bicinchoninic acid (BCA), chitosan (CS), hollow filaments β-TPC/SrCuSi_4_O_10_ (hTCP/SC), human bone marrow mesenchymal stem cells (BMSC), human umbilical vein endothelial cells (HUVEC), methotrexate (MTX), nano-hydroxyapatite (nHA), perfluorotributylamine (PFA), polydopamine (PDA), polydopamine (PDA), β-tricalcium phosphate (β-TPC), radioimmunoprecipitation assay (RIPA), tirapazamine (TPZ), zeolitic imidazolate frameworks (ZIF-8). * PTA: Photothermal agents.

### 3.4. Graphene Oxide in Drug Delivery

Graphene oxide, due to its amphiphilic properties, produces van der Waals-type interactions and π–π stacking with different organic materials that allow the adsorbing of many polar polymers, making it an excellent material for forming GO/polymer compounds; in addition, it exhibits fluorescence in visible and near-infrared regions [175,176]. These compounds cause molecules deposited on the surface of GO to be immobilized, making these nanocomplexes excellent candidates as drug carriers [177]. The immobilization of molecules is effective because active oxygenated groups, such as hydroxyls, carboxyl, ether, epoxide, quinone, and lactone, are present on the surface of the GO (Figure 3). These groups have hydrophilic characteristics, good biocompatibility, and strong interactions with proteins, metals, drugs, cells, and biomolecules. This is due to covalent bond formation, π–π stacking, hydrogen bonding, hydrophobic interactions, and the electrostatic force [164]. Rajaei et al. reported that nanocomposites based on chitosan, agarose, and graphene oxide (CS/AG/GO) hydrogels loaded with 5-fluorouracil (5-FU) were prepared by the water-oil-water (W/O/W) emulsification technique, exhibiting a loading and encapsulation efficiency of 57% and 92%, respectively, compared to mesoporous silica nanoparticles as drug carriers; the nanocarriers released 5-FU in a more effective and controlled way at a pH of 5.4 within 48 h. Its cytotoxicity was assessed in breast cancer cells (MCF-7) incubated with CS/AG/GO, showing a 23% increase in the effectiveness of its anticancer capacity [178].

Li et al. used a dual-response drug delivery system (DDS) to treat osteosarcomas, varying the pH and near-infrared light exposure, NIR [179,180]. It was reported that graphene oxide was loaded with naringin (Nar) and co-encapsulated with methotrexate (MTX) to be incorporated into an oxidized alginate hydrogel (OxAlg) and carboxymethyl chitosan (CMCS) via a Schiff base reaction. By being at pH 5.0 and exposed to NIR, an acceleration in drug administration was achieved, demonstrating that the system released up to 91.09% of MTX and 85.69% of Nar, in addition to generating an increase in the treatment of osteosarcoma [181]. Graphene oxide as a drug delivery system is a promising field of research towards the application of cancer, presenting improvements in efficacy, increasing yield, and minimizing drug toxicity through the controlled release of factors such as pH and temperature.

### 3.5. Nanocarriers in Cancer Treatment

**Figure 7:**
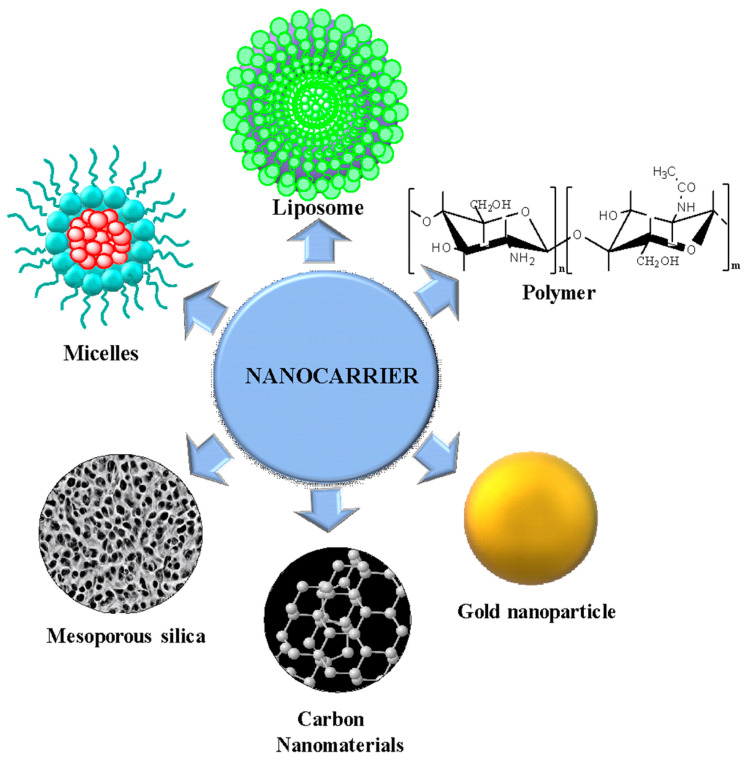
Outline of the types of nanocarriers used in cancer therapy.

Drugs given for cancer treatment are limited for different reasons, such as drug resistance, poor selectivity, solubility, poor efficiency, and the sensitivity of tumor cells [182]. In the case of osteosarcoma, obstructing the bone marrow decreases blood flow, making it challenging to administer anticancer drugs. Therefore, with the use of nanocarriers, new alternatives that allow a reduction of these side effects are sought [183]. When combined with drugs, nanoparticles are more effective and more specific than the combination of multiple medications, avoiding drug resistance [184]. Nanocarriers are an excellent alternative because they release drugs in varying temperatures, pH [185], and under magnetism [186], in addition to providing selectivity and many other advantages. There are different types of nanocarriers for cancer treatment, such as organic nanocarriers such as polymers [187], liposomes [188], micelles [189], and dendrimers [190]. Inorganic nanocarriers such as metallic NPs are also found [191], as are porous silica nanomaterials [192], calcium phosphate carriers [193], and carbon-based nanomaterials [194] (Figure 7).

Graphene has excellent physicochemical properties and acceptable biocompatibility. Unlike graphene, graphene oxide has hydrophobic regions and hydrophilic edges that give it good aqueous dispersibility and the ability to cross cell membranes easily and allow biological molecules to associate with it, facilitating efficient drug loading [92]. Graphene oxide nanocarriers can disperse in biological systems due to their hydrophilic characteristics, creating interactions with enzymes, peptides, proteins, and other components. When interacting with proteins, it forms a hard layer called the protein crown; this coating can induce changes in GO size, which in turn influences its interactions with cells, such as its biodistribution, biocompatibility, therapeutic efficacy, and biodegradation [195,196]. The study of protein adsorption on the surface of graphene oxide influences the behavior and fate of GO.

Molecular mechanisms have studied the adsorption of proteins on the surface of graphene; for example, Ortega et al. described, through molecular dynamic simulation with explicit and implicit solvents, the adsorption of immunoglobulin G on the surface of graphene [197]. In the explicit solvent, the protein travels from the solution to the material’s surface by a diffusion mechanism driven by electrostatic or hydrophobic interactions. The water–water interaction gain balances the water–protein loss, thus regulating the electrostatic contribution of solvation energy. The van de Waals force between them compensates for this breakdown of the solvation layers around the protein and the surface. Despite the energy compensation, the protein is adsorbed on the surface of the graphene. On the other hand, in the implicit solvent, they underestimate the cost of breaking down the solvation layers of both the protein and the substrate, resulting in instant adsorption and protein deployment. We conclude that the driving force given in the adsorption process is not only enthalpic but also entropic in origin, in addition to the implicit solvent methods, which are insufficient to describe protein adsorption and proposed to correct specific characteristics of the implicit methods.

Therefore, it is necessary to look for strategies that allow for modifying the adsorption of proteins in graphene oxide as a nanocarrier. Functionalization in GO nanocarriers, then, is a strategy that uses covalent and non-covalent systems to reduce toxicity and improve the biocompatibility of graphene oxide. Covalent functionalization involves the derivatization of functional groups found on the material’s surface, such as hydroxyl, carboxyl, and epoxides, which are functionalized by amidation and esterification reactions. Non-covalent functionalization occurs through van der Waals interactions and π–π stacking [198]. Functionalization can be performed with biomolecules, peptides, enzymes, and proteins that prevent their opsonization or recognition by the immune system. Functionalization can also occur with biopolymers, such as chitosan, alginate, starch, and polymers, such as polyethylene glycol [199].

Kazemi et al. synthesized and characterized a curcumin nanocomposite using chitosan magnetite-reduced graphene oxide (Cur-CS-Fe_3_O_4_-RGO) as a nanocarrier in MCF-7 breast cancer cells [194]. Their study revealed that the double emulsion technique using chitosan and graphene oxide contributed significantly to a higher carrying capacity of curcumin, with a percentage of 63% and a trapping efficiency of 95.5%. In addition, they demonstrated an effective response to releasing 96% of curcumin at a pH of 5.4 after 72 h, compared to only 40% released at a pH of 7.4 during the same period. It was also observed that the nanocarrier Cur-CS-Fe3O4-RGO induced apoptosis in 33% of MCF-7 breast cancer cells. These findings suggest that the Cur-CS-Fe3O4-RGO nanocarrier has excellent potential as an effective treatment against MCF-7 cancer cells.

Carbon-derived nanomaterials such as carbon nanotubes (CNTs), carbon quantum dots (CQDs) [200], mesoporous carbon (MC), reduced graphene oxide (rGO), and graphene oxide are potential drug transporters [201,202]. In addition, GO can form complexes with drugs such as DOX through π–π stacking and hydrophobic interactions [203]. For example, Jeshvaghani et al. first employed the efficient dual nano-emulsification method to synthesize a novel nanocarrier of polyethylene glycol, graphene oxide, and natural silk fibroin protein, PEG/GO/SF/DOX, sensitive to pH changes [204]. It was found to increase the entrapment and charging efficiency by 87.75 ± 0.7% and 46 ± 1%, respectively, due to its high electrostatic capacity and the specific surface area of the GO. These values were attributed to the large specific surface capacity and functional groups of graphene oxide due to the forming of a π–π stack between the GO and the amine and hydroxyl groups of DOX, allowing a more significant amount of DOX to be loaded. The nanocomplex also achieved a cumulative and sustained release after 96 h, with release percentages of 95.75% and 90.25% in acidic and relatively neutral environments, respectively. An improvement in the inhibition of MCF-7 breast cancer cells by the nanocarrier was also obtained, demonstrating its potential for cancer treatment.

**Figure 8:**
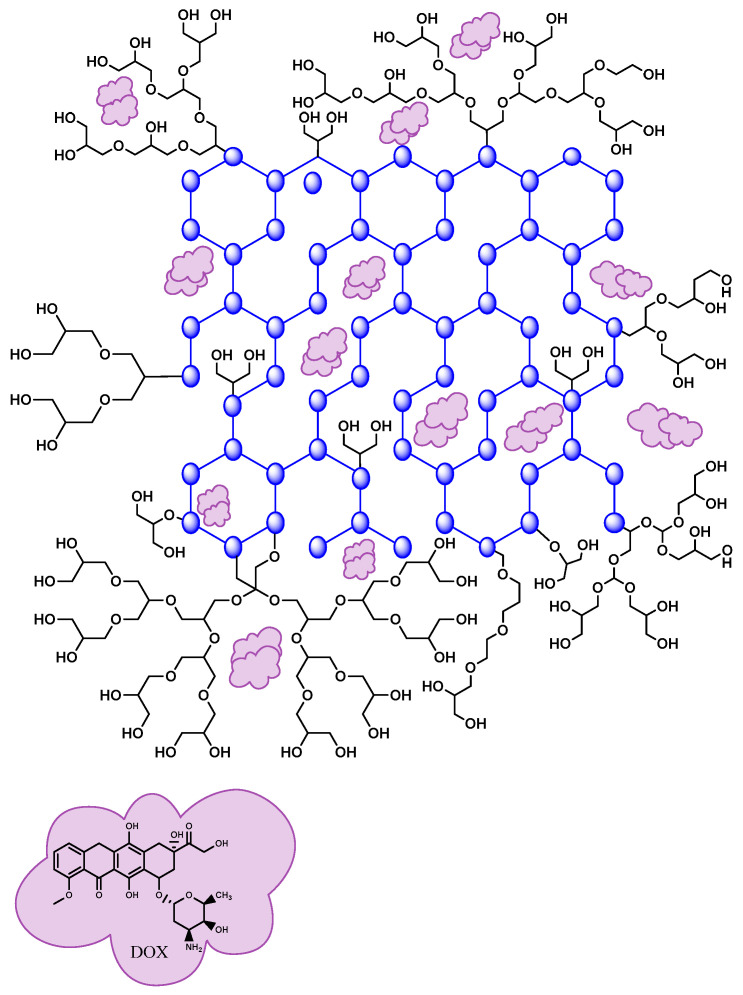
Schematic of the interaction of the GO nanocarrier with DOX-loaded polyglycerol grafts.

On the other hand, Targhazeh et al. electrostatically linked DOX to a graphene oxide nanocarrier with pH-sensitive polyhydroxyglycerol branch grafts (Figure 8) [205]. The study showed that the trisaminomethane modification provided abundant O-H and C=O groups and introduced nitrogen. Polyglycerol grafting improved the GO’s load capacity and encapsulation efficiency with values of 9.94% and 99.2%, respectively. In vitro release under various pH levels showed that in the first 6 h, at pH levels of 5.2 and 37 °C, more than 48% of DOX was rapidly released. After 14 days, the release was 78%, 70%, and 54% for pH = 5.2, pH = 6.8, and pH = 7.4, respectively. In addition, a 90.96% incorporation of the DOX nanocarrier by Saos-2 cells was achieved, and the absorption rate of free DOX was 99.94%, demonstrating that branched GO with polyglycerol is a potential carrier of antiproliferative drugs with high cellular toxicity, useful against cancer cells.

Graphene-based nanomaterials have shown great potential as nanocarriers in the treatment of osteosarcomas. However, functionalization through organic and inorganic pathways, biomolecules, and synthetic and natural polymers is essential to overcome their limitations. This process increases the stability and solubility of the nanomaterial in the biological medium, which can improve biocompatibility and mitigate toxicity [206].

## 4. Future Perspectives

Considering all the promising properties of GO shown for the treatment of bone cancer, there is a pressing need to improve the methods and formulations for GO functionalization. In this sense, future research can focus on GO functionalization strategies that allow more excellent physiological compatibilities to scale up to in vivo applications, as recently indicated by Taheriazam et al. [96]. Although the drugs used in the treatment of osteosarcomas represent an advance, they have side effects, a low solubility, and affect healthy tissue. There is a need to implement nanocarriers and therapies such as photodynamics and photothermal therapy, which offer novel and promising approaches to treating bone cancer. The functionalization of nanocarriers such as graphene oxide (GO), photothermal therapy (PTT), and photodynamic therapy (PTD) provide a synergistic effect, representing a promising advance to improve the therapeutic effects, specificity, solubility, and efficacy of the treatment of osteosarcomas. In the future, research should continue to explore the ability of light to penetrate deeper tissues, evaluating the effectiveness of treatments and their functionalization. In addition, it is crucial to deepen our investigation of the interactions between biomolecules and graphene derivatives to understand fundamental aspects such as biocompatibility, biodegradability, and pharmacokinetics. This is because there is research in various therapeutic areas, such as gene delivery, drugs, and photomedicine, but, until now, most of it has focused exclusively on graphene. Table 1, Table 2 and Table 3 summarize the anticancer applications of graphene oxide and reduced graphene oxide, as well as photodynamic and photothermal therapy, respectively. Satisfactory results have been presented in GO and rGO, primarily in vitro. However, future research should focus on in vivo applications and clinical studies, using more effective therapies rather than conventional ones. In addition, this highlights the need for future research to develop novel nanocarrier particles and combinations with other methods to overcome the limitations of photothermal therapy and the hypoxic tumor environment in photodynamic therapy.

## 5. Conclusions

This review article analyzes the current state of research related to the use of GO in treating osteosarcoma through an exhaustive systematic review and bibliometric analysis. It evaluates the existing literature on the subject. In this sense, studies focused on GO-based nanomaterials for applications against osteosarcoma were reviewed, which has revealed that there is an excellent trend toward the use of GO-based nanomaterials for the treatment of osteosarcoma through various therapeutic approaches, such as photothermal therapy, photodynamic therapy, and the use of nanocarriers. Photodynamic and photothermal therapy stand out as powerful therapeutic techniques for cancer treatment, overcoming limitations compared to other therapies. The combination of both therapies not only improves the delivery of photosensitizers but also achieves a synergistic effect. Additionally, the functionalization of therapies such as PTT, PTD, and nanocarriers generates positive effects in cancer treatment, overcoming existing treatments’ restrictions and achieving less invasiveness, and greater targeting and effectiveness. This review also highlights the recent advances of GO as a nanocarrier and its functionalization with biomolecules, resulting in a notable increase in the carrying capacity, trapping efficacy, and efficiency of chemotherapeutic agents. However, more research is needed to develop highly efficient localized therapies. On the other hand, the bibliometric analysis on the photothermal and photochemical treatment of graphene oxide and reduced graphene oxide in the supporting information, from January 2004 to May 2023, extracted 948 documents with its search strategy, mainly related to research papers, review papers, and conference papers, demonstrating a high-impact field supported by the need for more selective and efficient bone cancer therapies. The central countries leading the research are the United States, Iran, Italy, Germany, China, South Korea, and Australia, with strong collaborations worldwide. At the same time, the most cited papers were published in journals with impact factors of more than 6.0 (2022), with more than 290 citations. All the results presented here demonstrated the large impact of using graphene oxide and reduced graphene oxide in cancer bone treatments as a safe and alternative therapy that will continue growing in the following years.

## Figures and Tables

**Table 1:** Sample of anti-cancer studies of GO and rGO.

Human Osteosarcoma Cell Lines	Type of Study	Method of Analysis of Anti-Cancer Activity	Results	Ref
Saos-2 and MG-63	In vitro	Flow cytometry; Evaluation of apoptosis by staining with 4’,6-diamidino-2-phenylindole (DAPI); eBios-science Annexin V-FITC; Apoptosis detection kit.	The GO nanocarrier loaded or functionalized with DOX inhibited cell proliferation depending on the dose (between 10 and 1280 µg/mL) after 12 h. Additionally, a synergistic effect was evidenced in the inhibition of cancer cells when MLT was combined with DOX due to the regulation of the X-linked inhibitor of apoptosis (XIAP) and the catalytic subunit of human telomerase (hTERT).	[93]
Saos-2 and MG-63	In vitro	The MTT and ROS assay; Carrier biocompatibility; Gene expression with PCR; In vitro release with concentration and pH variation and adjustment to the Peppas model for drug release.	This study used GO as a nanocarrier functionalized with chitosan nanoparticles for siRNA delivery. The results of this study showed a controlled release of siRNA in the acidic pH prevailing at the tumor site. In addition, it inhibited the activity of the Bcl-2 oncogene (considered the main factor in multidrug resistance). This is interesting because it facilitates the treatment of the disease and prevents the infection from reappearing by inhibiting the overexpression of Bcl-2.	[94]
Saos-2	In vitro	Thermogravimetric analysis (TGA); Morphological characterization of the scaffolds by scanning electron microscopy (SEM); Uniaxial compression tests; Cell viability was assessed 1, 3, and 7 days after cell seeding using the Alamar Blue assay.	Osteosarcoma treatment using poly(ε-caprolactone)/graphene porous scaffolds obtained by liquid fusion with GO nanosheets demonstrated good mechanical properties and up to 83.5% inhibition of the disease. In addition, the results of this research show that the application of these scaffolds reduces the survival rate of cancer cells.	[100]
MG-63	In vivo	Flow cytometry was obtained with a Fortessa LSR; Identification of living and dead cells; Intracellular release of ROS; Western blot of precipitated components.	The non-covalent stable interaction of trastuzumab (TRA) with GO (TRA-GO) demonstrated good binding to the Her2 antibody (a potential therapeutic target), causing the rapid inhibition of cancer cells. In addition, TRA-GO induced oxidative stress and Her2 signaling in target cells; this induced the rapid depletion of cellular inhibitors of apoptosis protein (cIAP) and caspase8, RIP1/RIPK3/MLKL necroptosome formation, and necroptosis of cancer cells, significantly enhancing the antitumor activity of TRA.	[98]
MG-63	In vivo	Dialysis bag method to assess drug release in vitro; CCK-8 assays for the detection of viable cells; Colony formation number; Transwell assays for the analysis of cell migration and cell invasion Cellular production of ROS by 7’-dichlorodihydrofluorescein Di acetate staining; Detection of annexin V-FITC apoptosis; Flow cytometry; Assays for forming spheres—capturing the image visualized in a fluorescence microscope; Total protein using RIPA and BCA measurement; In vivo nasopharyngeal carcinoma cell tumor growth in nude mice.	The synthesis of GO-based nanoparticles doped with photosensitizers indocyanine green (ICG), folic acid (FA), and polyethylene glycol (PEG) and loaded with ginsenoside (Rg3: a significant component of ginseng), called PEG-GO-FA/ICG -Rg3, inhibited the proliferation, invasion, migration, and enhanced apoptosis and autophagy of cancer cells. This study demonstrated that PEG-GO-FA/ICG-Rg3 improves osteosarcoma cells’ tumor growth inhibition capacity, which presents a promising therapeutic strategy for treating osteosarcoma.	[101]
MC3T3-E1	In vitro	Flow cytometry; Cell size and complexity analysis; Cell viability was determined using flow cytometry; Effects of GO nanosheets on cell proliferation with a Neubauer Hemocytometer; Cell cycle analysis and apoptosis detection; Alkaline phosphatase activity; Alkaline phosphatase (ALP) activity was used as a marker to assess the expression of the osteoblastic phenotype; Matrix mineralization assay in cell cultures through alizarin red staining.	Functionalization of GO with polyethylene glycol-amine (PEG) significantly decreased cancer cells’ proliferation and increased their apoptosis. This study suggests that the application of the GO-PEG nanomaterial is promising at a concentration of 40 µg/mL since, at this concentration, it does not affect the differentiation of healthy preosteoclasts.	[102]
Saos-2	In vitro	Cell viability; Conventional method cell count kit 8 (CCK-8); Radiation method sensitive to pH, redox, and near-infrared (NIR); Chemophotothermal therapy.	A methotrexate (MTX) delivery system based on a mesoporous structure of @polidopamine@GO silica nanoparticles improved the drug delivery capacity and photothermal capacity for chemo-photothermal applications of osteosarcoma.	[103]
U2OS and Saos-2	In vitro	CRISPR-CAS9; Western blotting; Cell morphology; eBios-science annexin apoptosis detection kit; Detection of reactive oxygen species.	Induction of apoptosis in cancer cells via GO and CRISPR-Cas9. This study targets the IGF1 and IGFBP3 signaling pathways, strengthening GO-related cytotoxicity.	[104]

Abbreviations: 3-(4,5-Dimethylthiazol-2-yl)-2, 5-diphenyl tetrazolium bromide (MTT), 4’,6-diamidino-2-phenylindole (DAPI), Alkaline phosphatase (ALP), catalytic subunit of human telomerase (hTERT), cell counting kit-8 (CCK-8), cellular inhibitors of apoptosis protein (cIAP), doxorubicin (DOX), folic acid (FA), graphene oxide (GO), indocyanine green (ICG), insulin growth factor 1 (IGF1), insulin growth factor binding protein (IGFBP3), loaded with ginsenoside (Rg3: a significant component of ginseng), melatonin (MLT), methotrexate (MTX), near-infrared (NIR), polyethylene glycol-amine (PEG), polymerase chain reaction (PCR), reactive oxygen species (ROS), scanning electron microscopy (SEM), short interfering RNA (siRNA), the human epidermal growth factor receptor 2 (HER2), thermogravimetric analysis (TGA), trastuzumab (TRA), X-linked inhibitor of apoptosis (XIAP).

**Table 2:** Different types of photodynamic therapy (PDT) systems in applications against osteosarcomas.

PDT System	PS	Type of Study	Method of Analysis of Anti-Cancer Activity of Osteosarcoma	Results	Ref
Poly(lactic-co-glycolic acid) nanoparticles were concealed with human osteosarcoma cell membranes and encapsulated with the photosensitizer IR780 (MH-PLGA-IR780).	IR780	In vivo, In vitro	Human OS cells HOS, MG63, and 143b. Double emulsion method W/O/W. CLSM focal laser scanning microscopy. CCK-8 cell viability. TEM and ROS DCFH-DA, Western blot measurement. In vivo study in mice with HOS. 808 nm NIR laser.	Greater penetration into deeper tissues and apoptosis and ferroptosis induction were achieved. In addition, an absorption rate of more than 90% of MH-PLGA-IR780 generates a high affinity with the HOS cell line. Intracellular ROS percentages of 98.97% were obtained, improving PDT performance.	[130]
Bovine serum albumin nanoparticles—zinc phthalocyanine (BSA-ZnPc, BZ)	ZnPc	In vivo, In vitro	Male mice weighing (16–18 g) MNNG/HOS and MNNG/HOS-Luc. Tumor necrosis factor ELISA kit. Apoptosis was assessed by fluorescence-activated cell classification (FACS) and flow cytometry. To detect ROS in cells, DCFH-DA ROS assay kits were used.	BSA as a ZnP carrier increased water solubility and enhanced PDT effects. An increase in tumor resection and a more significant effect of PDT were achieved without affecting healthy tissue. In addition, results were obtained on the cytotoxicity of BZ after irradiation and the reliability of cells when exposed to BZ without irradiation.	[128]
Chemophotodynamically functionalized graphene oxide (GO) nanoparticles (PEG-GO-FA/ICG).	ICG	In vitro, In vivo	808 nm NIR laser. Human osteosarcoma cells (MNNG/HOS, U2OS, MG63, and SaOS-2) and cell viability with CCK-8 assay. For the detection of ROS in cells: DCFH-DA ROS assay. Flow cytometry and Western blot. For the analysis of tumor tissue: hematoxylin-eosin staining.	It was found that the PEG-GO-FA/ICG had a more significant photothermal effect as the temperature increased over periods, remaining above 50 °C. A loading yield of 54% of the drugs and a stability of 30% of the in vitro release of the PEG-GO-FA/ICG nanoparticles (DOX + TH287) at an acidic pH were obtained. Cells with a chemo-photodynamic effect showed high calcium levels in the cytoplasm of osteosarcoma cells, showing more significant damage to the ER membrane.	[129]
Poly(ethylene glycol)-poly [2(methyl acryloyl)ethylnicotinate] nanoparticles with Zinc phthalocyanine (PEG-PMAN/ZnPc) (PPZ).	ZnPc	In vitro, In vivo	PE/Annexin V apoptosis detection kit. Irradiation with RL (1.8 kJ/cm^2^, 660 nm. ROS oxidation kit.	ROS production was achieved in MNNG/Hos osteosarcoma cells, in addition to obtaining a charge content of 8.2%, an encapsulation efficiency of 89.4%, and an extended absorption at a wavelength of 660 nm, favorable for PTD and improved proliferation inhibition in the in vitro study. Significant tumor growth inhibition was achieved after 14 days of 21.7 mm^3^, with PPZ being a therapeutic potential for PDT in osteosarcomas.	[131]

Abbreviations: bovine serum albumin-Zinc phthalocyanine (BSA-ZnPc; BZ), confocal laser scanning microscopy (CLSM), dichlorodihydrofluorescein diacetate (DCFH-DA), endoplasmic reticulum (ER), fluorescence-activated cell sorting (FACS), folic acid (FA), (HOS) cell membrane human osteosarcoma, osteosarcoma (OS), PEG-PMAN/ZnPc nanoparticle (PPZ), photodynamic therapy (PDT), poly (ethylene glycol)-poly[2-(methylacryloyl)ethylnicotinate] (PEG-MAN), poly (lactic-co-glycolic acid) (PLGA), transmission electron microscopy (TEM), water-oil-water (W/O/W), Zinc phthalocyanine (ZnPc).

## Data Availability

Data from the bibliometric analysis are available from the corresponding author upon request.

## Supplementary Materials

The following supporting information can be downloaded at https://www.mdpi.com/article/10.3390/nano14020186/s1. The selected publications’ imported data (publication and authors) were obtained from Scopus as an Excel file. All the bibliometric analysis figures and tables can also be observed in Figure S1, as can the number of publications per year (2010–2023) related to graphene oxide and bone cancer. Figure S2; the map of co-occurrence network visualization based on article weights for the terms associated with the first group. Figure S3; co-authorship network of countries publishing on graphene oxide and bone cancer topics (threshold of 33). Table S1; The top 20 most cited articles (2010–2023). Figure S4; citation network diagram of documents from articles mentioned a minimum of 50 times. Table S2; The top 10 publishing sources on graphene oxide and reduced graphene oxide applications for cancer therapy. Figure S5; co-citation network diagram of journals from which articles have been cited a minimum of 20 times. Refs. [67,103,152,207,208,209,210,211,212,213,214,215,216,217,218,219,220,221,222,223,224,225,226] are cited in Supplementary Materials.
