# Supplementary material for: Graphene Oxide (GO) for the Treatment of Bone Cancer: A Systematic Review and Bibliometric Analysis
Source: Nanomaterials (Basel). 2024 Jan 13;14(2):186. doi: 10.3390/nano14020186 (PMC10820493; doi:10.3390/nano14020186)
Supplement: Supplementary file 1 [file nanomaterials-14-00186-s001.zip › nanomaterials-2776374-supplementary.pdf]

nanomaterials

Support information

Graphene oxide (GO) for treating bone cancer: A systematic review and bibliometric analysis

Lemy Vanessa Barba-Rosado[1], Domingo César Carrascal-Hernández[1,2], Daniel Insuasty[2] and Carlos David Grande-Tovar[1,*]

[1] Grupo de Investigación en Fotoquímica y Fotobiología, Programa de Química, Facultad de Ciencias Básicas, Universidad del Atlántico, Puerto Colombia 081008, Colombia
[2] Departamento de Química y Biología, División de Ciencias Básicas, Universidad del Norte, Km 5 Vía Puerto Colombia, Barranquilla 081007, Colombia
* Correspondence: carlosgrande@mail.uniatlantico.edu.co ; Tel.: +57-5-3599484

Abstract: Cancer is a severe disease that, in 2022, caused more than 9.89 million deaths worldwide. One worrisome type of cancer is bone cancer, such as osteosarcoma and Ewing tumors, which occur more frequently in infants. This study shows an active interest in using graphene oxide and its derivatives in therapy against bone cancer. We present a systematic review analyzing the current state of the art related to the use of GO in treating osteosarcoma through evaluating the existing literature. In this sense, studies directed at GO-based nanomaterials for potential applications against osteosarcoma were reviewed, which has revealed that there is an excellent trend toward the use of GO-based nanomaterials, based on their thermal and anti-cancer activities for the treatment of osteosarcoma through various therapeutic approaches. However, more research is needed to develop highly efficient localized therapies. The bibliometric analysis on photothermal and photochemical treatment of graphene oxide and reduced graphene oxide from January 2004 to December 2022 extracted 948 documents with the search strategy, mainly related to research papers, review papers, and conference papers, demonstrating a high-impact field supported by the need of more selective and efficient bone cancer therapies. The central countries leading the research are the United States, Iran, Italy, Germany, China, South Korea, and Australia, with stronger collaborations worldwide. At the same time, the most cited papers were published in journals of impact factors of more than 6.0 (2021), with more than 290 citations. Besides, the journals that published the most on the topic are high impact factor Journals, according to the analysis performed, demonstrating the high impact of the research field.

Keywords: graphene oxide; reduced graphene oxide; osteosarcoma.

1. Bibliometric analysis

1.1. How many publications are produced per year on the topic?

It is well known that Bibliometric analysis maps academic literature and quantitatively assesses trends in those research topics [1,2]. The bibliometric study on photothermal and photochemical therapy of graphene oxide and reduced graphene oxide is an active and recent topic. The 948 documents extracted with the search strategy demonstrate that this is a high-impact field supported by the need for more selective and efficient bone cancer therapies. After the refined data explained in the methodology, 873 documents were extracted. Of them, 652 (68.8%) correspond to research articles, 289 (30.5%) to review articles, and seven conference papers (0.7%). The analysis shows that the publications began in 2010 (2), while the number of publications per year increased almost linearly up to 2020, when the tendency began to plateau (Figure S1). This might be due to the COVID-19 pandemic emergency that the world experimented on between the end of 2019 and

May 2023, which restricted the experimental research. The number of publications between 2020 (120), 2021 (131), and 2022 (121) confirms the trend. However, it might also be possible that the topic is suffering a transition from basic research to applied research, combining methodologies and technology for bone cancer treatment. For example, a dual drug delivery system with triple response (response to pH changes, near-infrared (NIR) irradiation, and redox) was proposed based on mesoporous silica nanoparticles@polydopamine@graphene oxide nanosheets for chemo photothermal therapy against osteosarcoma using the encapsulation of methotrexate (MTX) and naringin [3]. This study shows that multiple drugs and technologies are combined for the most effective treatment of osteosarcoma, seeking to complement the treatments and avoid cell resistance to drugs. Besides, 2023 is not finished yet; the trend looks like the last three years, with 53 documents published until the date of the search (June 4, 2023), indicating that the topic reached a plateau and is suffering a transition to applications in the technology field. Several patents might result in the next couple of years supporting this statement.

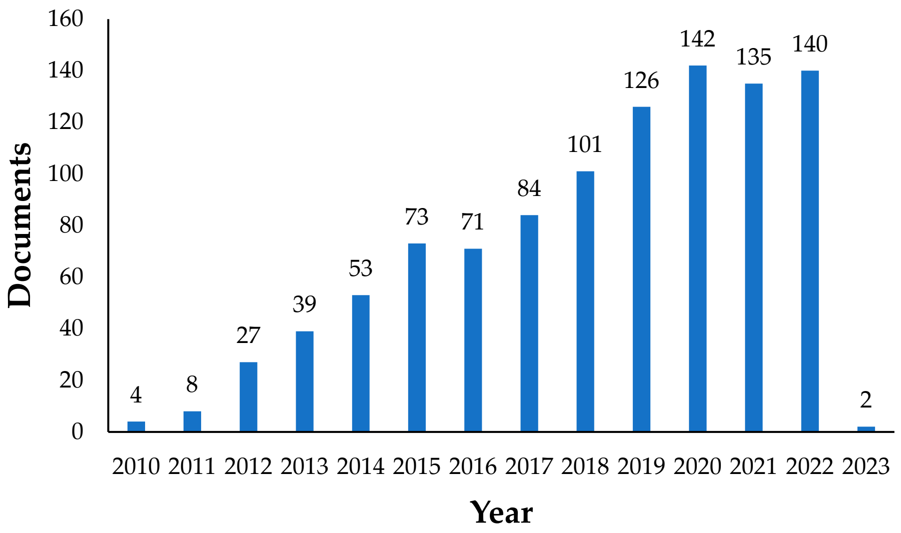

Figure S1. The number of publications per year (2010–2023) related to graphene oxide and bone cancer.

1.2. What are the most relevant keywords in the GO and rGO treatment of bone cancer?

According to the analysis using the VOSviewer software, the most critical keywords cited at least ten times (based on the node's size and the strength of the link with other keywords) correspond to 669 words grouped into five clusters, with 118923 links and a total strength of 559700. It is crucial to notice that general terms such as "human," "nonhuman," and "article," while terms duplicated indicating only plural were removed. Cluster 1 has the most important keyword related to "drug delivery system" with 661 links, a total link strength of 13446, and 337 occurrences, indicating the importance of studying different nanocarriers and drugs for chemical cancer therapy, such as doxorubicin and paclitaxel, as well as the use of multiple systems based on nanomaterials for imagen and biosensing, using compounds such as the indocyanine green. It is also interesting to see the second cluster, where the term "graphene" is the essential term, with 662 links, a total link strength of 15359, and 508 occurrences, also demonstrating a good connection and proximity with terms such as "reduced graphene oxide," "infrared devices," "diseases," "chemotherapy," and "photothermal therapy," indicating the use of these graphene oxide derivatives for combined therapies against bone and breast cancer, primarily based on photothermal treatment. The third cluster has "graphene oxide" as the

primary term, with 663 links, 22142 total link strength, and 589 occurrences. This cluster is more related to in vivo and in vitro studies, cell viability, controlled studies, cytotoxicity, and antineoplasic studies, using graphene oxide as a nanocarrier, mainly for doxorubicin, for cancer chemotherapy. Cluster 4 only includes six items related to biocompatibility studies, tissue distribution, and disease models. Cluster five has only three items, which include "neoplasm" and "photothermal," indicating a possible new area of study related to the effect of photothermal therapy in neoplasms.

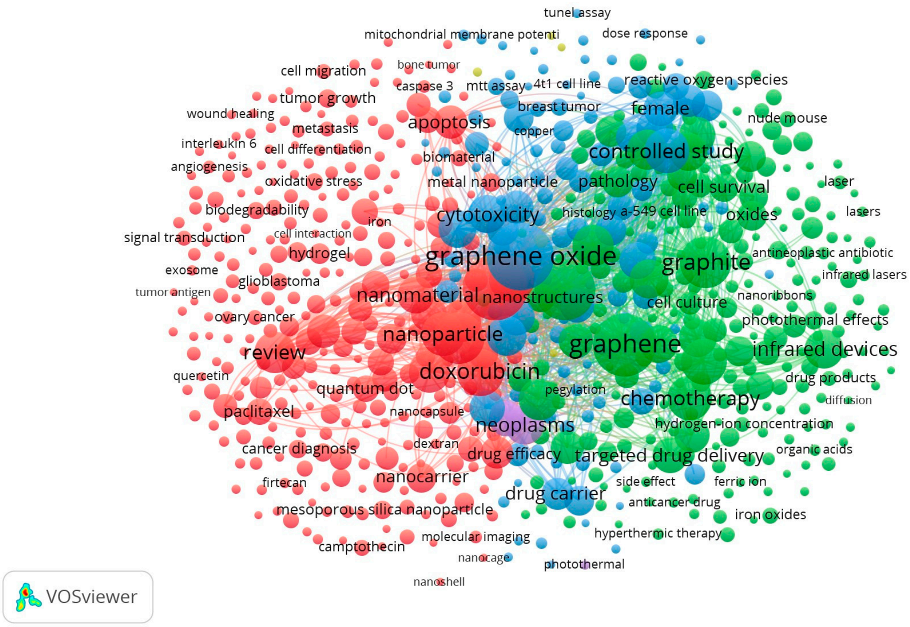

Figure S2. Map of co-occurrence network visualization based on article weights for the terms related to the first group. Items:664, links: 118923, total link strength of 559700. Term relationships are shown as curved lines of varying thickness determined by co-occurrence. Their relationship determines the distance between items. The term size is determined by its occurrence.

1.3. What is the collaboration between countries on this issue?

There are seven clusters indicating the collaboration between the countries on this topic. Iran is the most important country in the first cluster, with 22 links and a total link strength of 90 and 98 documents. It is close to Turkey but reflects a solid collaboration with several countries worldwide. The Second cluster is represented by India, with 23 links, a total link strength of 67, and 81 documents. India has a strong collaboration with the United States (link strength of 7) but also with China (link strength of 6) and Saudi Arabia (link strength of 13), which are Asian countries. The third cluster is represented by South Korea, with 17 links, a total strength of 39, and 76 documents. The main collaborations of South Korea are China (link strength 9) and India (link strength 5). Cluster 4 has two countries representing the main collaborations—China, with 22 links, 134 total strengths, and 453 documents. China has a robust partnership with the United States (link strength 42), the other representative country in the cluster, with 23 links, 122 total strengths, and 114 documents. Australia represents the fifth cluster with 20 links, a real strength of 52 and 27 papers. The main collaborations of Australia are China (link strength 14), Italy – (link strength 5), and Iran (link strength 4). However, global collaboration is particularly interesting for this country. The sixth cluster is represented by Germany, with 12 links, a total strength of 27 and 19 documents, and a paramount collaboration with the

central partnerships with China (link strength 6), Iran (link strength 5), and the United States (link strength 3). Finally, the seventh cluster is represented by Italy with 19 links, a total strength of 36 and 30 documents, and a strong collaboration between Iran (link strength 5) and Australia (link strength 5). Besides Brazil, no South American countries are on the map, indicating a lack of research published in this field. Moreover, South Africa is the only African country in the analysis, meaning that the leading countries contributing to the research in this field are European, Asian, Oceanic, and North American.

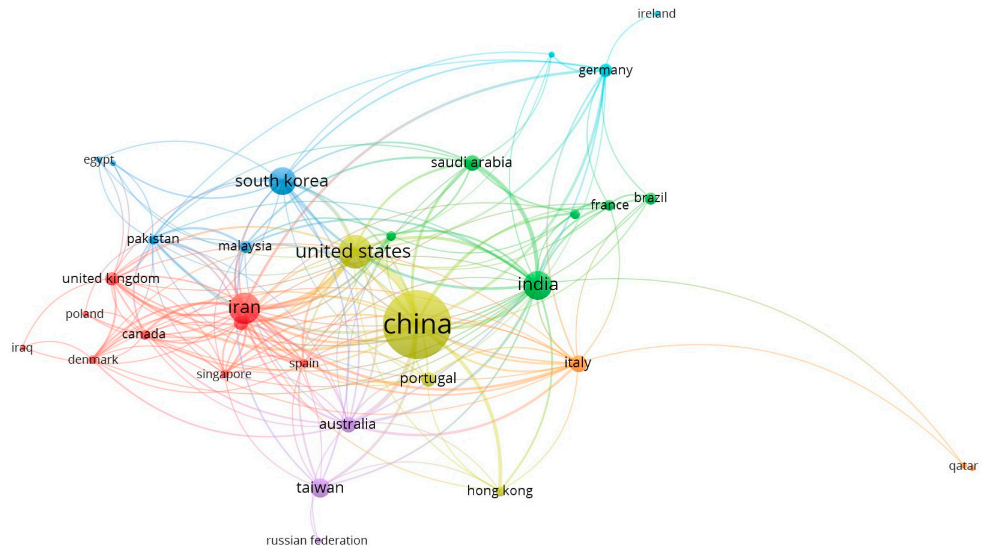

Figure S3. Co-authorship network of countries from graphene oxide and bone cancer topic (threshold of 33). The size of the nodes indicates the number of publications produced by the country. The proximity of the two nodes indicates the relatedness of their co-authorship link, while the thickness of the connecting line indicates the strength of the cooperation. Items: 33. Clusters: 7. Links: 189. Total link strength: 479.

1.4. What are the most cited articles?

From the total articles extracted (873), 211 (24.1%) have at least 50 citations or more, indicating the topic's relevance in the scientific literature. Besides Table S1 contains the Top 20 most cited articles on the subject, all the journals from those articles have high impact factors (2022) and Cite Scores (2022), such as the *Chemical Society Reviews* (Cite Score of 81.3 and a Journal impact factor of 46.2) journal. The three most cited articles included in Table S1 correspond to *Graphene in Mice: Ultrahigh in Vivo Tumor Uptake and Efficient Photothermal Therapy* with 2114 citations [4]. *Ultrasmall Reduced Graphene Oxide with High Near-Infrared Absorbance for Photothermal Therapy* with 1776 citations [5], and *Functional Graphene Oxide as a Nanocarrier for Controlled Loading and Targeted Delivery of Mixed Anti-cancer Drugs* with 1522 citations [6]. All these studies are the pioneer studies using graphene oxide and reduced graphene oxide for photothermal and nanocarrier in cancer therapy, which also reflects the importance of the topic by the number of citations and the journal's impact factor and Cite Score of the journals where those articles were published.

Table S1. The Top 20 most cited documents (2010 – 2023)

Total citations	Cite Score 2022	Journal's Impact Factor 2022	Reference
2114	18.0	10.8	[4]
1776	25.7	15.0	[5]
1522	19.0	13.3	[6]
1379	81.3	46.2	[7]
980	17.0	9.7	[8]
841	45.5	29.4	[9]
788	23.4	14.0	[10]
683	23.4	14.0	[11]
676	81.3	46.2	[12]
614	20.9	12.4	[13]
543	13.7	9.9	[14]
542	45.5	29.4	[15]
542	19.0	13.3	[16]
376	N/A	6.626	[17]

356	10.1	4.7	[18]
319	19.0	13.3	[19]
313	23.4	14.0	[20]
305	23.4	14.0	[21]
298	25.4	17.1	[22]
290	21.5	13.6	[23]

Figure S3 also demonstrates the most cited papers, confirming the trend observed in Table S1. However, it is also relevant the proximity of each node indicates the similarity in the studies from the topic and the connections between the Top 3 studies, which are placed at the center of the network, indicating the relevance of the subject for all the literature published up today in the field.

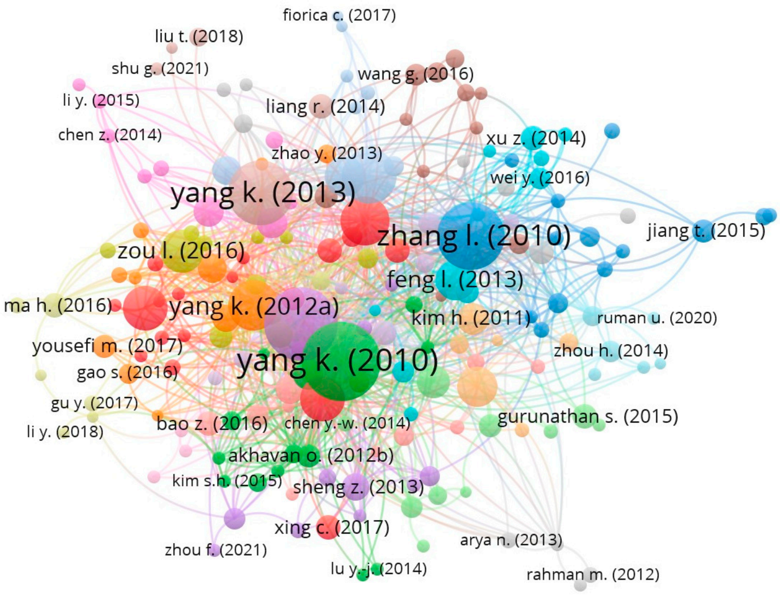

Figure S4. Citation network diagram of documents from articles cited a minimum of 50 times. Items: 188. Clusters: 21. Links: 939.

1.5. What are the journals that are most involved in the field?

Table S2 shows the Top 10 journals that publish the most on the topic, with at least 15 documents or more. The articles published in the Top 10 journals account for at least 24.7% of the total articles extracted during the search. According to the Clarivate Journal Citation Report, all those ten journals work in the common areas of materials' properties, pharmaceutical nanocarriers, photochemistry, controlled release of pharmaceuticals, and surface chemistry, all essential properties in the design of photo and chemical therapy agents.

Table S2. The Top 10 publishing sources on graphene oxide and reduced graphene oxide applications for cancer therapy.

Journal Title	Number of documents	Cite Score 2022	Journal's Impact Factor 2022
Journal of Materials Chemistry B	32	11.5	7.0
Biomaterials	30	23.4	14.0
International Journal of Nanomedicine	28	14.0	8.0
ACS Applied Materials and Interfaces	21	15.7	9.5
Pharmaceutics	21	6.9	5.4
Colloids and Surfaces B-Biointerfaces	19	11.0	5.8
International Journal of Molecular Sciences	18	7.8	5.6
Journal of Photochemistry and Photobiology B Biology	16	13.4	5.4
Materials Science and Engineering C (Until 2022)	16	12.6 (2021)	8.457 (2021)
Journal of Controlled Release	15	17.1	10.8

Figure S4 exhibits the map of co-citations between the journals constructed with VOSviewer. It is well known that the thickness of the links between the two nodes (journals) represents the strength of the co-citation between two journals, while the node size means the journal's total documents.

As can be seen, the Top 10 journals from Table S2 have the most significant node sizes and the thickest links, indicating the highest number of documents and co-citations between the journals. Besides, the Top five journals from Table S2 are also in the center of the 4 clusters, which also reflects the importance of the journal in the topics and the closest of the subjects' areas.

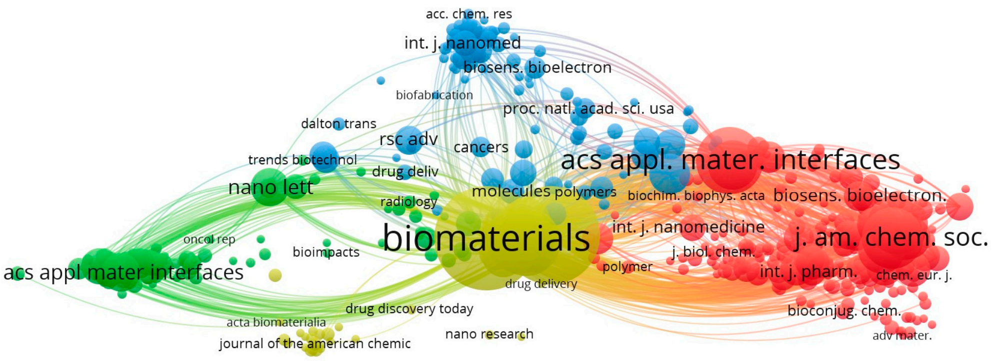

Figure S5. Co-citation network diagram of journals from articles cited a minimum of 20 times. Items: 484. Clusters: 4. Links: 60482. Total link strength: 2380599.

References

1. Zhang, Y.; Pu, S.; Lv, X.; Gao, Y.; Ge, L. Global Trends and Prospects in Microplastics Research: A Bibliometric Analysis. *J. Hazard. Mater.* **2020**, *400*, 123110, doi:https://doi.org/10.1016/j.jhazmat.2020.123110.
2. Jiang, Y.; Xia, W.; Zhao, R.; Wang, M.; Tang, J.; Wei, Y. Insight into the Interaction Between Microplastics and Microorganisms Based on a Bibliometric and Visualized Analysis. *Bull. Environ. Contam. Toxicol.* **2021**, doi:10.1007/s00128-021-03201-y.
3. Gao, J.; Cao, C.; Rui, Q.; Sheng, Y.; Cai, W.; Li, J.; Kong, Y. A Tri-Responsive Dual-Drug Delivery System Based on Mesoporous Silica Nanoparticles@ Polydopamine@ Graphene Oxide Nanosheets for Chemo-Photothermal Therapy of Osteosarcoma. *J. Saudi Chem. Soc.* **2023**, 101655.
4. Yang, K.; Zhang, S.; Zhang, G.; Sun, X.; Lee, S.T.; Liu, Z. Graphene in Mice: Ultrahigh in Vivo Tumor Uptake and Efficient Photothermal Therapy. *Nano Lett.* **2010**, *10*, 3318.
5. Robinson, J.T.; Tabakman, S.M.; Liang, Y.; Wang, H.; Sanchez Casalongue, H.; Vinh, D.; Dai, H. Ultrasmall Reduced Graphene Oxide with High Near-Infrared Absorbance for Photothermal Therapy. *J. Am. Chem. Soc.* **2011**, *133*, 6825–6831, doi:10.1021/ja2010175.
6. Zhang, L.; Xia, J.; Zhao, Q.; Liu, L.; Zhang, Z. Functional Graphene Oxide as a Nanocarrier for Controlled Loading and Targeted Delivery of Mixed Anticancer Drugs. *Small* **2010**, *6*, 537.
7. Yang, K.; Feng, L.; Shi, X.; Liu, Z. Nano-Graphene in Biomedicine: Theranostic Applications. *Chem. Soc. Rev.* **2013**, *42*, 530–547, doi:10.1039/C2CS35342C.
8. Liu, J.; Cui, L.; Losic, D. Graphene and Graphene Oxide as New Nanocarriers for Drug Delivery Applications. *Acta Biomater.* **2013**, *9*, 9243–9257, doi:10.1016/j.actbio.2013.08.016.
9. Yang, K.; Hu, L.; Ma, X.; Ye, S.; Cheng, L.; Shi, X.; Li, C.; Li, Y.; Liu, Z. Multimodal Imaging Guided Photothermal Therapy Using Functionalized Graphene Nanosheets Anchored with Magnetic Nanoparticles. *Adv. Mater.* **2012**, *24*, 1868–1872, doi:10.1002/adma.201104964.
10. Zhang, W.; Guo, Z.; Huang, D.; Liu, Z.; Guo, X.; Zhong, H. Synergistic Effect of Chemo-Photothermal Therapy Using PEGylated Graphene Oxide. *Biomaterials* **2011**, *32*, 8555–8561, doi:10.1016/j.biomaterials.2011.07.071.
11. Yang, K.; Wan, J.; Zhang, S.; Tian, B.; Zhang, Y.; Liu, Z. The Influence of Surface Chemistry and Size of Nanoscale Graphene Oxide on Photothermal Therapy of Cancer Using Ultra-Low Laser Power. *Biomaterials* **2012**, *33*, 2206–2214, doi:10.1016/j.biomaterials.2011.11.064.
12. Shanmugam, V.; Selvakumar, S.; Yeh, C.-S. Near-Infrared Light-Responsive Nanomaterials in Cancer Therapeutics. *Chem. Soc. Rev.* **2014**, *43*, 6254–6287, doi:10.1039/C4CS00011K.
13. Zou, L.; Wang, H.; He, B.; Zeng, L.; Tan, T.; Cao, H.; He, X.; Zhang, Z.; Guo, S.; Li, Y. Current Approaches of Photothermal Therapy in Treating Cancer Metastasis with Nanotherapeutics. *Theranostics* **2016**, *6*, 762–772, doi:10.7150/thno.14988.
14. Ma, X.; Tao, H.; Yang, K.; Feng, L.; Cheng, L.; Shi, X.; Li, Y.; Guo, L.; Liu, Z. A Functionalized Graphene Oxide-Iron Oxide Nanocomposite for Magnetically Targeted Drug Delivery, Photothermal Therapy, and Magnetic Resonance Imaging. *Nano Res.* **2012**, *5*, 199–212, doi:10.1007/s12274-012-0200-y.
15. Feng, L.; Wu, L.; Qu, X. New Horizons for Diagnostics and Therapeutic Applications of Graphene and Graphene Oxide. *Adv. Mater.* **2013**, *25*, 168–186, doi:10.1002/adma.201203229.
16. Zhang, L.; Lu, Z.; Zhao, Q.; Huang, J.; Shen, H.; Zhang, Z. Enhanced Chemotherapy Efficacy by Sequential Delivery of siRNA and Anticancer Drugs Using PEI-Grafted Graphene Oxide. *Small* **2011**, *7*, 460–464, doi:10.1002/smll.201001522.
17. Akhavan, O.; Ghaderi, E.; Aghayee, S.; Fereydooni, Y.; Talebi, A. The Use of a Glucose-Reduced Graphene Oxide Suspension

for Photothermal Cancer Therapy. *J. Mater. Chem.* **2012**, *22*, 13773.

18. Kim, H.; Namgung, R.; Singha, K.; Oh, I.-K.; Kim, W.J. Graphene Oxide–Polyethylenimine Nanoconstruct as a Gene Delivery Vector and Bioimaging Tool. *Bioconjug. Chem.* **2011**, *22*, 2558–2567, doi:10.1021/bc200397j.
19. Akhavan, O.; Ghaderi, E. Graphene Nanomesh Promises Extremely Efficient in Vivo Photothermal Therapy. *Small* **2013**, *9*, 3593–3601, doi:10.1002/smll.201203106.
20. Wang, Y.; Wang, H.; Liu, D.; Song, S.; Wang, X.; Zhang, H. Graphene Oxide Covalently Grafted Upconversion Nanoparticles for Combined NIR Mediated Imaging and Photothermal/Photodynamic Cancer Therapy. *Biomaterials* **2013**, *34*, 7715–7724, doi:10.1016/j.biomaterials.2013.06.045.
21. Sahu, A.; Choi, W.I.; Lee, J.H.; Tae, G. Graphene Oxide Mediated Delivery of Methylene Blue for Combined Photodynamic and Photothermal Therapy. *Biomaterials* **2013**, *34*, 6239–6248, doi:10.1016/j.biomaterials.2013.04.066.
22. Song, J.; Yang, X.; Jacobson, O.; Lin, L.; Huang, P.; Niu, G.; Ma, Q.; Chen, X. Sequential Drug Release and Enhanced Photothermal and Photoacoustic Effect of Hybrid Reduced Graphene Oxide-Loaded Ultrasmall Gold Nanorod Vesicles for Cancer Therapy. *ACS Nano* **2015**, *9*, 9199–9209, doi:10.1021/acsnano.5b03804.
23. Shibu, E.S.; Hamada, M.; Murase, N.; Biju, V. Nanomaterials Formulations for Photothermal and Photodynamic Therapy of Cancer. *J. Photochem. Photobiol. C Photochem. Rev.* **2013**, *15*, 53–72, doi:https://doi.org/10.1016/j.jphotochemrev.2012.09.004.

Disclaimer/Publisher's Note: The statements, opinions, and data contained in all publications are solely those of the individual author(s) and contributor(s) and not of MDPI and/or the editor(s). MDPI and/or the editor(s) disclaim responsibility for any injury to people or property resulting from any ideas, methods, instructions or products referred to in the content.
